# Functionalization of Brain Region-specific Spheroids with Isogenic Microglia-like Cells

**DOI:** 10.1038/s41598-019-47444-6

**Published:** 2019-07-30

**Authors:** Liqing Song, Xuegang Yuan, Zachary Jones, Cynthia Vied, Yu Miao, Mark Marzano, Thien Hua, Qing-Xiang Amy Sang, Jingjiao Guan, Teng Ma, Yi Zhou, Yan Li

**Affiliations:** 1 Department of Chemical and Biomedical Engineering, FAMU-FSU College of Engineering, Florida State University, Tallahassee, FL USA; 2 Department of Biomedical Sciences, College of Medicine, Florida State University, Tallahassee, Florida USA; 3 The Translational Science Laboratory, College of Medicine, Florida State University, Tallahassee, Florida USA; 4 Department of Chemistry and Biochemistry, Florida State University, Tallahassee, Florida USA; 5 Institute of Molecular Biophysics, Florida State University, Tallahassee, Florida USA; 6 Present Address: Department of Chemical Engineering, Carnegie Mellon University, Pittsburgh, Pennsylvania USA

**Keywords:** Tissue engineering, Stem-cell biotechnology

## Abstract

Current brain spheroids or organoids derived from human induced pluripotent stem cells (hiPSCs) still lack a microglia component, the resident immune cells in the brain. The objective of this study is to engineer brain region-specific organoids from hiPSCs incorporated with isogenic microglia-like cells in order to enhance immune function. In this study, microglia-like cells were derived from hiPSCs using a simplified protocol with stage-wise growth factor induction, which expressed several phenotypic markers, including CD11b, IBA-1, CX3CR1, and P2RY12, and phagocytosed micron-size super-paramagnetic iron oxides. The derived cells were able to upregulate pro-inflammatory gene (TNF-α) and secrete anti-inflammatory cytokines (i.e., VEGF, TGF-β1, and PGE2) when stimulated with amyloid β42 oligomers, lipopolysaccharides, or dexamethasone. The derived isogenic dorsal cortical (higher expression of TBR1 and PAX6) and ventral (higher expression of NKX2.1 and PROX1) spheroids/organoids displayed action potentials and synaptic activities. Co-culturing the microglia-like cells (MG) with the dorsal (D) or ventral (V) organoids showed differential migration ability, intracellular Ca^2+^ signaling, and the response to pro-inflammatory stimuli (V-MG group had higher TNF-α and TREM2 expression). Transcriptome analysis exhibited 37 microglia-related genes that were differentially expressed in MG and D-MG groups. In addition, the hybrid D-MG spheroids exhibited higher levels of immunoreceptor genes in activating members, but the MG group contained higher levels for most of genes in inhibitory members (except SIGLEC5 and CD200). This study should advance our understanding of the microglia function in brain-like tissue and establish a transformative approach to modulate cellular microenvironment toward the goal of treating various neurological disorders.

## Introduction

Human induced pluripotent stem cells (hiPSCs) have shown great potential to generate physiologically relevant neural cells^1^, tissues and spheroids (*i.e*., brain organoids)^2–13^ for studying neurological disease progression and virus infections (*e.g*., Zika)^14–18^. *In vitro* brain organoids can be used to probe early stages of disease on-set for preventative therapeutics or for drug screening^8,19,20^. Recently, the fusion of brain organoids of dorsal or ventral regions provides a feasible approach to study interneuron (GABAergic) migration and heterotypic cell-cell interactions^21–23^. However, current brain organoids have several issues: (1) lack vascularization and microglia cells in specific organoid regions^24^; (2) immaturity of neuronal cells; (3) cell type diversity and reproducibility (batch effects and local microenvironment effects)^25^. Among these issues, microglia cells play an important role in early cortical development and neurological disorder progression^26^. Significant knowledge gaps still exist due to the lack of interactions of neural cells with microglia^18^. So the immune response and neuroprotective capacity of hiPSC-derived brain organoids are still limited^27^.

Microglia are resident macrophage-like cells in human brain and account for 5–20% of total neural cells in parenchyma^28–30^. They have two main functions: immune defense and the maintenance and development of the central nervous system^28,29^. Microglia restrain potential damage to the central nervous system and support tissue repair and remodeling, under the inflammatory conditions of an active immune response. They can phagocytosis pathogens and cell debris, removing toxic molecules and protein deposits^31^. Moreover, microglia controls and regulates synaptic plasticity through pruning, during its development^32^. As tissue resident immune cells, microglia are originated from primitive hematopoietic stem cells at yolk sac stage which migrate to the developing neural tube^33^. Due to unique origin, primary human microglia are not easily accessible. A major clinical hurdle of understanding pathology of brain disorders is the lack of abundant amount of normal or diseased microglia cells. The recently developed brain organoids from hiPSCs mostly lack the component of microglia^24^. Thus, fully mimicking the function of human brain has not been achieved.

Recently, a few studies derived microglia-like cells or precursors from hiPSCs^17,33–39^. The results demonstrate the similarity of hiPSC-derived microglia-like cells with human microglia, suggesting that generating microglia from hiPSCs is possible. However, most microglia derivations lack of brain-region-specific microenvironment and how microglia function in different brain regions is not well known. For example, forebrain microglia but not cerebellar microglia depend on IL-34 for maintenance^40^. Given the brain region-dependent microglia diversity^40,41^, it is believed that brain region-specific microenvironment promotes microglia function, and mutually microglia show selective regional sensitivity with neural cells. Microglia dysfunction has been found for various diseases such as Alzheimer’s disease, Parkinson’s disease, schizophrenia, and brain tumors^35,37,42^. Understanding the roles of microglia in human brain tissue development is expected to greatly facilitate the construction of complex 3D brain-like organoids, thereby advancing neurological disease modeling and drug screening^43,44^.

Our previous studies investigated different patterning factors in Wnt and sonic hedgehog (SHH) signaling to control brain-like tissue regional identity, the bioreactor systems in cortical spheroid formation, and the influence of mesenchymal stem cells on dorsal cortical spheroid formation from hiPSCs^45–50^. Built on our previous studies, it is hypothesized that incorporation of microglia-like cells enhances immune response ability of cortical spheroids/organoids with different regional identity, i.e., dorsal or ventral. The novelty of this study in comparison to previous study is that microglia-like cells are integrated with the dorsal or ventral organoids rather than whole cerebral organoids^34,51^. So the objectives of this study include: (1) to generate and characterize microglia-like cells from hiPSCs using a simplified protocol; (2) to investigate the impacts of microglia-like cells on the microenvironment of 3D region-specific cortical organoids with dorsal or ventral identity; (3) to investigate the impacts of microglia-like cells on attenuating neural inflammation in the “hybrid” organoids. This study should advance our understanding of microglia function in brain-like tissue and establish a transformative approach to modulate cellular microenvironment for treating various neurological disorders through novel drug discovery.

## Results

### Derivation and characterizations of microglia-like cells from human iPSK3 cells

**Figure 1:**
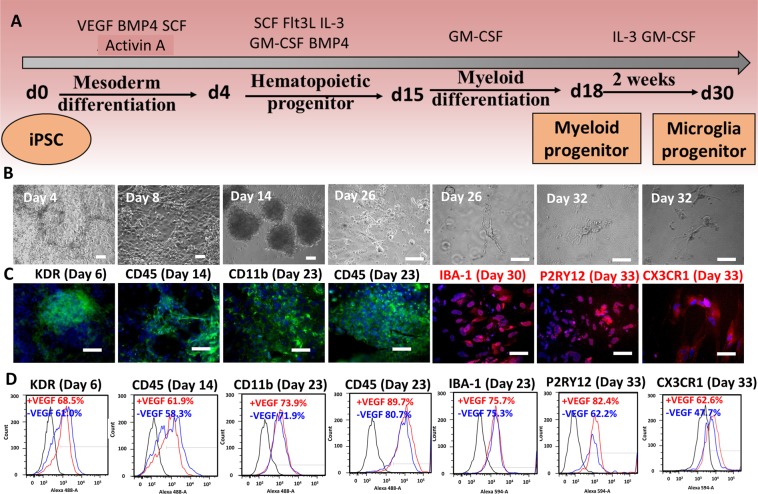
Induction of microglia-like cells from hiPSCs. (**A**) Schematic illustration of the derivation of microglia precursors from hiPSCs. (**B**) Phase contrast images of the derived microglia-like cells over 30 days. (**B**) Representative fluorescent images of derived mesoderm progenitors, hematopoietic progenitors, and microglia-like progenitors for KDR, CD45, CD11b, IBA-1, CX3CR1, and P2RY12. Blue: Hoechst 33342. Scale bar: 100 μm; (**D**) Representative flow cytometry histograms of microglia-like cells expressing KDR, CD45, CD11b, IBA-1, CX3CR1, and P2RY12 at different stages. Black line: negative control; red line: differentiation in the presence of VEGF, blue line: differentiation in the absence of VEGF.

The protocol to derive microglial-like cells from hiPSCs is shown in Fig. 1A. The hiPSCs were induced into hematovascular mesoderm lineage with the differentiation medium containing BMP-4, Activin-A, SCF and VEGF. VEGF presence (+VEGF) and absence (−VEGF) conditions were compared in order to elucidate the role of VEGF in microglia (MG) derivation. KDR^+^ mesoderm progenitors appeared by day 5–6 (Fig. 1B,C). CD45^+^ (a pan-leukocyte marker) myeloid progenitors were detected after 14 days in differentiation. The CD45 expression increased with time and reached 89% by day 23. In addition, the derived MGs expressed high levels of CD11b (integrin α_M_ and a marker for mature myeloid cells, also known as Mac-1) and IBA-1 (a cytoplasmic calcium-binding protein) by day 23 (Fig. 1C,D). After 30 days, myeloid progenitors differentiated into microglial progenitors, expressing IBA-1, P2RY12 (a purinergic receptor), and CX3CR1 (Fig. 1C,D). Further differentiation resulted in microglia-like ramified morphology by day 35–40. About 2–3 microglia-like cells were generated per input iPSK3 cell.

**Figure 2:**
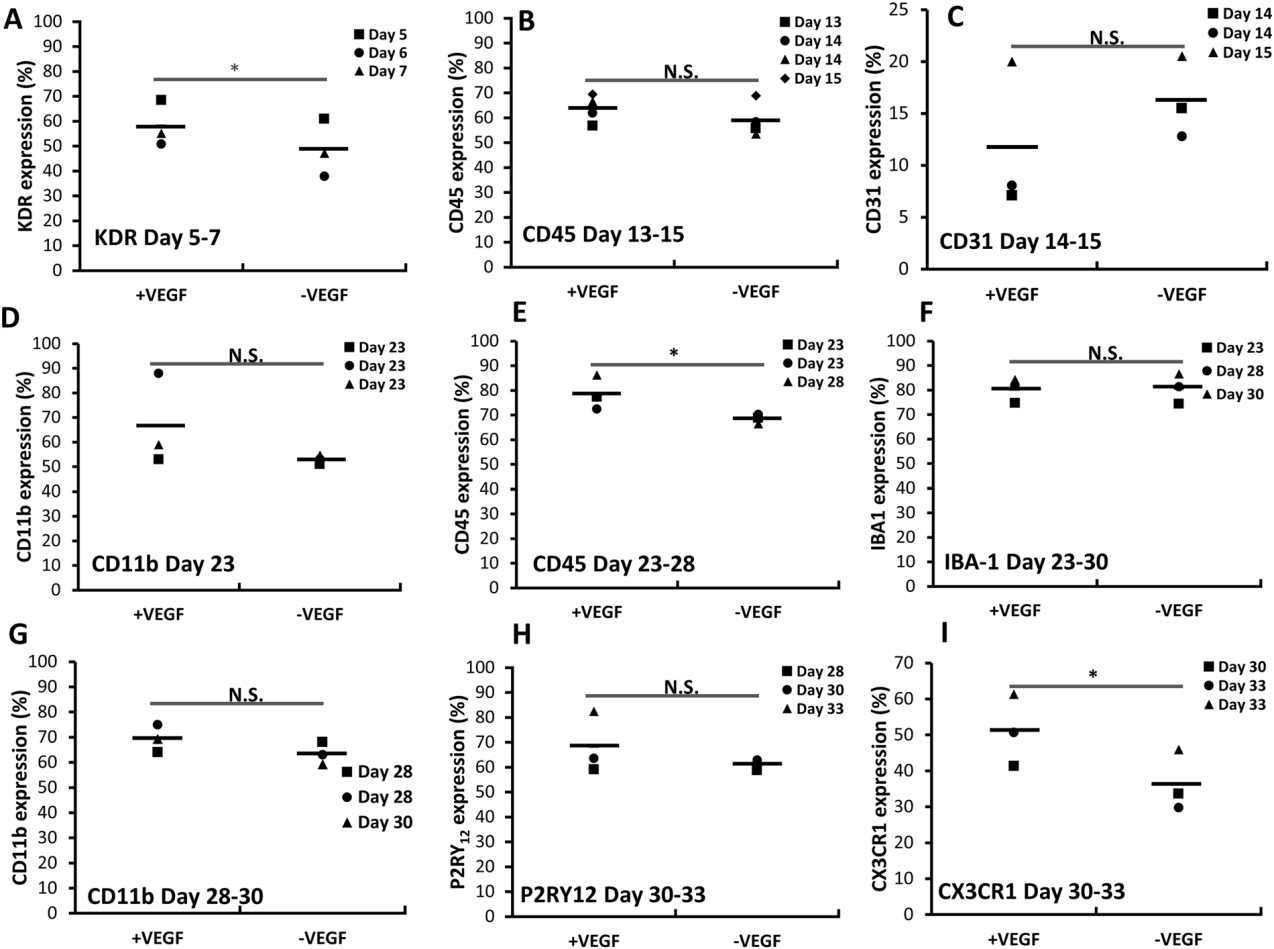
Phenotype characterizations at different stages of microglia-like cell differentiation. Cell phenotype was characterized by flow cytometry for at least three derivations (n = 3 or 4) for differentiation in the presence of (+) VEGF or in the absence of (−) VEGF. (**A**) KDR (day 5–7). Day 13–15 samples: (**B**) CD45; (**C**) CD31. Day 23–30 samples: (**D**) CD11b, (**E**) CD45, and (**F**) IBA-1. Day 28–33 microglial markers, (**G**) CD11b, (**H**) P2RY12, (**I**)) CX3CR1. *Indicates *p* < 0.05 for the different test conditions.

Microglia phenotype was analyzed by flow cytometry for three or four independent derivations (Fig. 2). The expression of KDR (day 5–7), CD45 (day 23–28), and CXCR1 (day 30–33) was higher for +VEGF group compared with −VEGF group. A low expression level of CD31 was observed. From day 13 to day 28, the expression of CD45 increased from 63.7 ± 5.5% to 78.7 ± 7.0% (Fig. 2B,E), while CD11b expression remained similar (66.6 ± 18.7% vs. 69.5 ± 5.5%) (Fig. 2E,G). After 30 days, the cells expressed more mature microglia markers IBA-1 (80.3 ± 4.9%, day 28–30) and P2RY12 (68.4 ± 12.3%, day 30–33), which are important for microglial homeostatic function (Fig. 2H)^52^. The derived cells also expressed CX3CR1 (51.1 ± 10.0%, day 30–33) (Fig. 2I), which plays an important role in neurogenesis and synaptic pruning by regulation of neuron-microglia interaction through CX3CL1-CX3CR1 signaling^53,54^. Microglial differentiation was also performed using another human iPSC line, Ep-iPSC, which was derived from CD34+ cord blood using a three-plasmid, seven-factor (SOKMNLT; SOX2, OCT4 (POU5F1), KLF4, MYC, NANOG, LIN28, and SV40L T antigen) EBNA-based episomal system (Supplementary Fig. S1).

### Functional analysis of microglia-like cells derived from human iPSK3 cells

**Figure 3:**
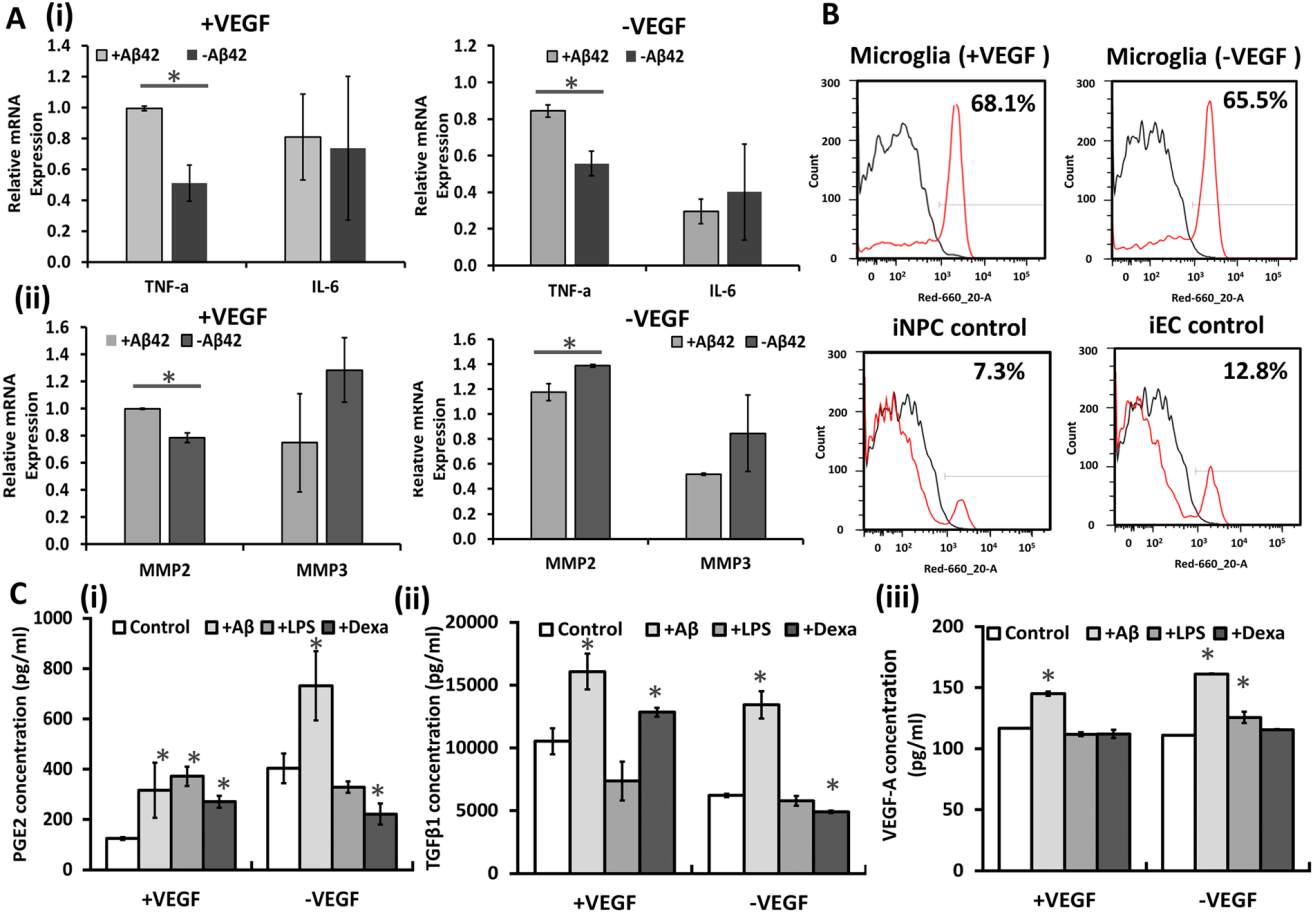
Functional characterizations of the derived microglia-like cells. (**A**) mRNA gene expression of (i) TNF-α, IL-6, and (ii) MMP2, MMP3 after Aβ42 oligomer stimulation for 72 hours (n = 3). *Indicates *p* < 0.05 for the different test conditions. (**B**) Quantification of micron-sized particles of iron oxide (MPIO) phagocytosis by microglia-like cells and iPSC-NPC (neural progenitor cells) and iPSC-EC (endothelial cells) controls (called as iNPC and iEC). Black line: negative control; red line: cells with MPIO. (**C**) The cytokine secretion of microglia-like cells (n = 3), stimulated by Aβ42 oligomers (0.5 µM), Lipopolysaccharides (LPS) (100 ng/ml), and dexamethasone (1 µM) for 72 hours as determined by ELISA assays. (i) PGE2; (ii) TGF-β1 and (iii) VEGF-A. *Indicates *p* < 0.05 for the test conditions compared to the control.

The ability of the derived MGs responding to pro-inflammatory, e.g., Aβ42 oligomers and lipopolysaccharides (LPS), and anti-inflammatory (e.g., dexamethasone) stimuli was examined^55^. Aβ42 oligomer treatment upregulated the inflammatory gene TNF-α expression (e.g., from 0.51 ± 0.12 to 0.99 ± 0.0 for +VEGF group) (Fig. 3A). Recent studies indicate that matrix metalloproteinases (MMPs) can regulate the activities of various pro-inflammatory cytokines, including TNF-α and IFN-γ^56,57^. Upregulation of MMP-2 was also observed for +VEGF group upon Aβ42 oligomer stimulation (Fig. 3A). The derived microglial-like cells phagocytosed more fluorescent micron-sized particles of iron oxide (MPIO) (65.5%-68.1%) than iNPC (7.3%) and iEC (12.8%) control groups (Fig. 3B and Supplementary Fig. S2). Subsequently, the secretion of anti-inflammatory cytokines responding to Aβ42 oligomers, LPS, and dexamethasone was compared (Fig. 3C and Supplementary Fig. S3). Microglial-like cells upregulated the secretion of PGE2, TGF-β1, and VEGF-A with Aβ42 oligomer treatment compared to the control. The LPS-activated microglial-like cells increased the secretion of PGE2 (for +VEGF group only) or VEGF-A (for −VEGF group only), while for the stimulation with dexamethasone, the cells secreted more of PGE2 and TGF-β1 (for +VEGF group only) compared to the control.

### Derivation and characterizations of dorsal and ventral spheroids and organoids

**Figure 4:**
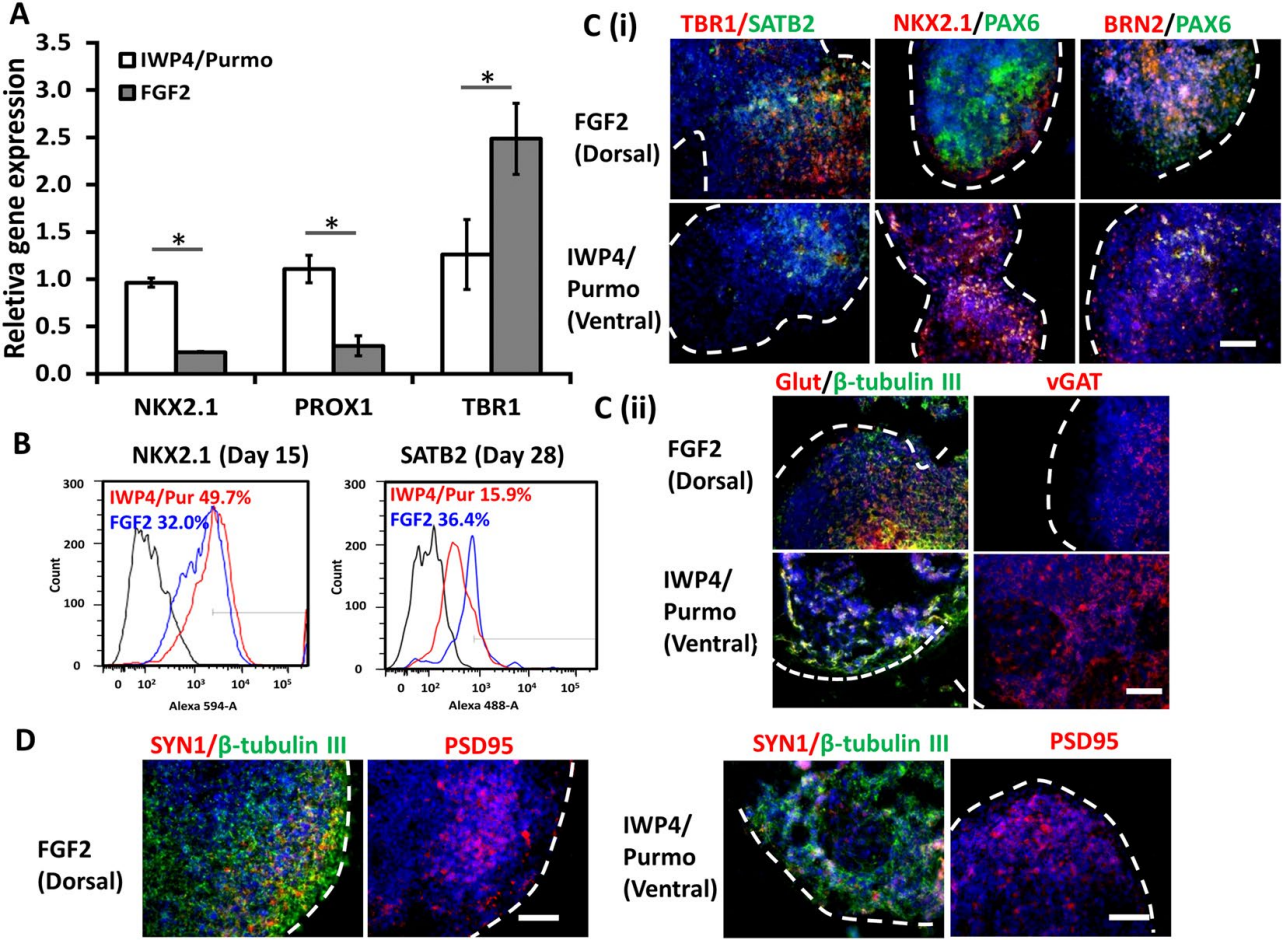
Phenotypic characterizations of day 47 dorsal and ventral organoids derived from hiPSCs. (**A**) mRNA expression of NKX2.1, PROX1, and TBR1 of dorsal and ventral organoids (n = 3). *Indicates *p* < 0.05 for the different test conditions. (**B**) Quantification of ventral neuron marker NKX2.1 and cortical layer II-IV marker SATB2 by flow cytometry. Black line: negative control. (**C**) (i) Representative images of histology sections for cortical layer markers TBR1 (red)/SATB2 (green), BRN2 (red)/PAX6 (green), ventral neuron markers, NKX2.1 (red)/PAX6 (green). (ii) Glutamatergic neuron marker, Glut (red)/β-tubulin III (green), and GABAergic neuron marker, vGAT (red). Blue: Hoechst 33342. Scale bar: 100 μm. (**D**) Representative images of histology sections for pre-synaptic marker synapsin I (red)/β-tubulin III (green) and post-synaptic marker PSD95 (red). Blue: Hoechst 33342. Scale bar: 100 μm. The dashed lines indicate the locations of spheroids.

Dorsal and ventral forebrain spheroids (<30 days) or organoids (>30 days) were derived in suspension (Supplementary Fig. S4). After 7 days of dual-SMAD inhibition, the spheroids were treated with fibroblast growth factor 2 (FGF2) to generate dorsal spheroids (referred as FGF2 group). The treatments of Wnt inhibitor IWP4 and the SHH agonist Purmorphamine generated ventral spheroids (referred as IWP4/Purmo group). RT-PCR analysis showed that IWP4/Purmo group had higher expression of ventral markers NKX2.1 (0.96 ± 0.05 vs. 0.23 ± 0.01) and PROX1 (1.11 ± 0.15 vs. 0.29 ± 0.11), while the FGF2 group had higher TBR1 (dorsal cortical marker) (2.49 ± 0.38 vs. 1.26 ± 0.37) (Fig. 4A). From flow cytometry, the FGF2 group had higher SATB2 (superficial cortical layer II-IV marker) expression than the IWP4/Purmo group (36.4% vs. 15.9%) at day 28 (Fig. 4B). By day 47, cells from the FGF2 group highly expressed TBR1 and SATB2 (Fig. 4C and Supplementary Fig. S5). The presence of late-born neurons (BRN2 expression) also confirmed the cortical identity (Fig. 4C). By contrast, the IWP4/Purmo group had higher NKX2.1 expression. Both groups had glutamatergic neurons as well as GABAergic neurons indicated by vGAT and GABA expression. The cells from both groups expressed pre-synaptic marker Synapsin I and post-synaptic marker PSD95 (Fig. 4D). Collectively, these results indicate that the FGF2 group enriches cells of dorsal cortical identity, while the IWP4/Purmo group enriches cells of ventral forebrain identity.

**Figure 5:**
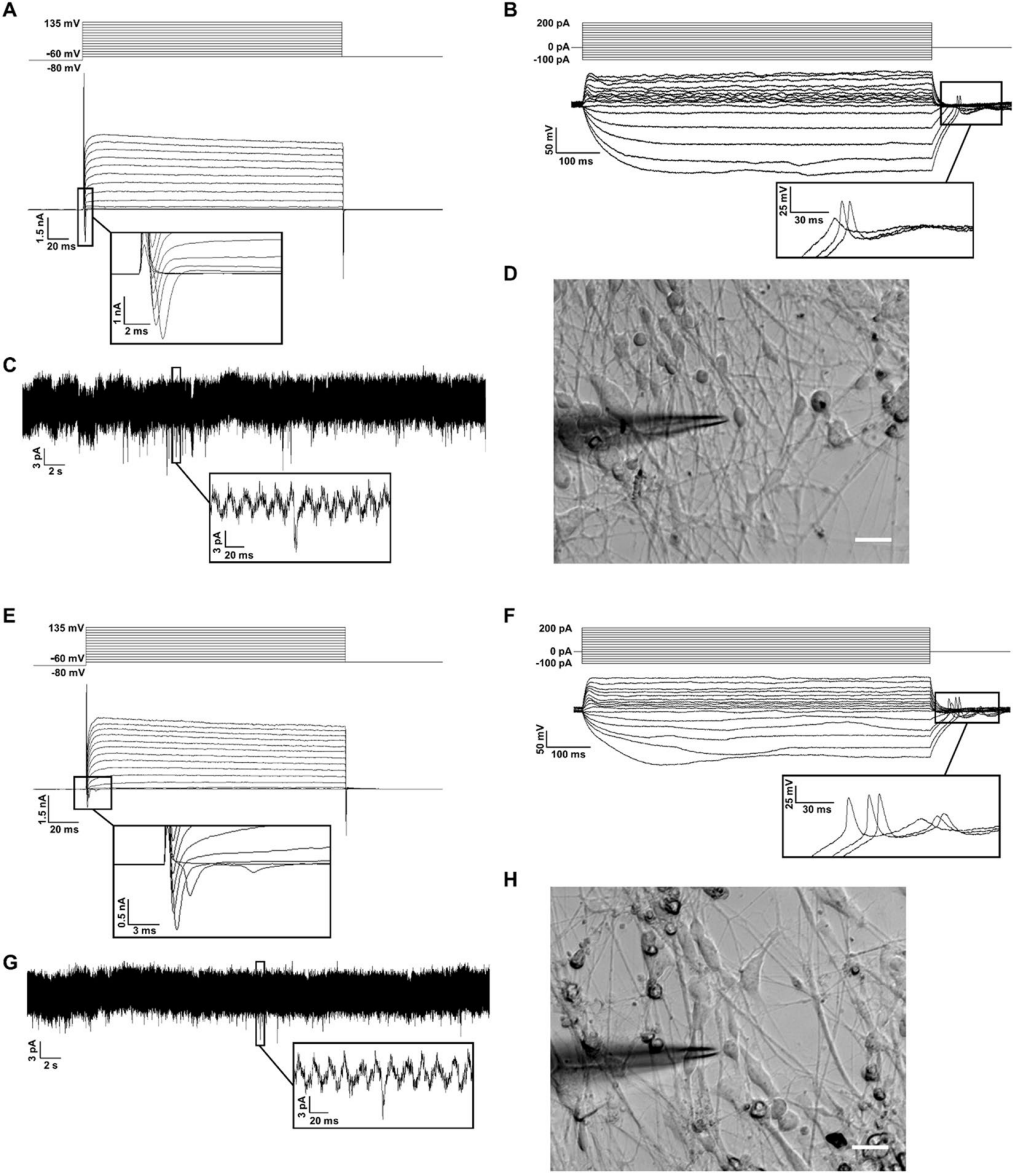
Electrophysiological properties of dorsal (**A–D**) and ventral (**E–H**) spheroids (day 45). (**A**,**E**) Representative voltage clamp traces showing fast inward Na+ currents and long-lasting outward K+ currents evoked by depolarizing voltage steps. Step size = 15 mV. The inset shows a high magnification view of the inward currents. (**B**,**F**) Representative current clamp traces showing rebound action potentials in response to hyperpolarizing current injections. Step size = 20 pA. The inset shows a high magnification view of the rebound action potentials. (**C,G**) Representative traces of continuous voltage clamp recording showing spontaneous postsynaptic currents. The inset shows a high magnification view of one spontaneous postsynaptic current. (**D**,**H**) Representative phase-contrast images of a recorded neuron. Scale bar: 20 μm.

The electrophysiological properties of the outgrowth cells of the derived organoids were examined via patch clamping. Both dorsal and ventral organoids displayed fast inward currents and long-lasting outward currents during voltage-clamp recording, suggesting the presence of functional voltage-gated Na^+^ and K^+^ channels, respectively (Fig. 5A,E). In addition, both spheroid groups fired rebound action potentials in response to hyperpolarizing current injection during current clamp recording (Fig. 5B,F). No action potential firing was observed during depolarizing current injection. Spontaneous postsynaptic currents were observed in the absence of stimulation during continuous voltage clamp recording (Fig. 5C,G). Cellular morphology of both spheroid types was stereotypically neuron-like, with small cell bodies and extensive projections (Fig. 5D,H). Together, these results suggest that the derived dorsal and ventral spheroids have the functional synaptic activities and the ability to fire action potentials.

### Co-culture of dorsal (D) and ventral (V) spheroids/organoids with microglia-like cells

**Figure 6:**
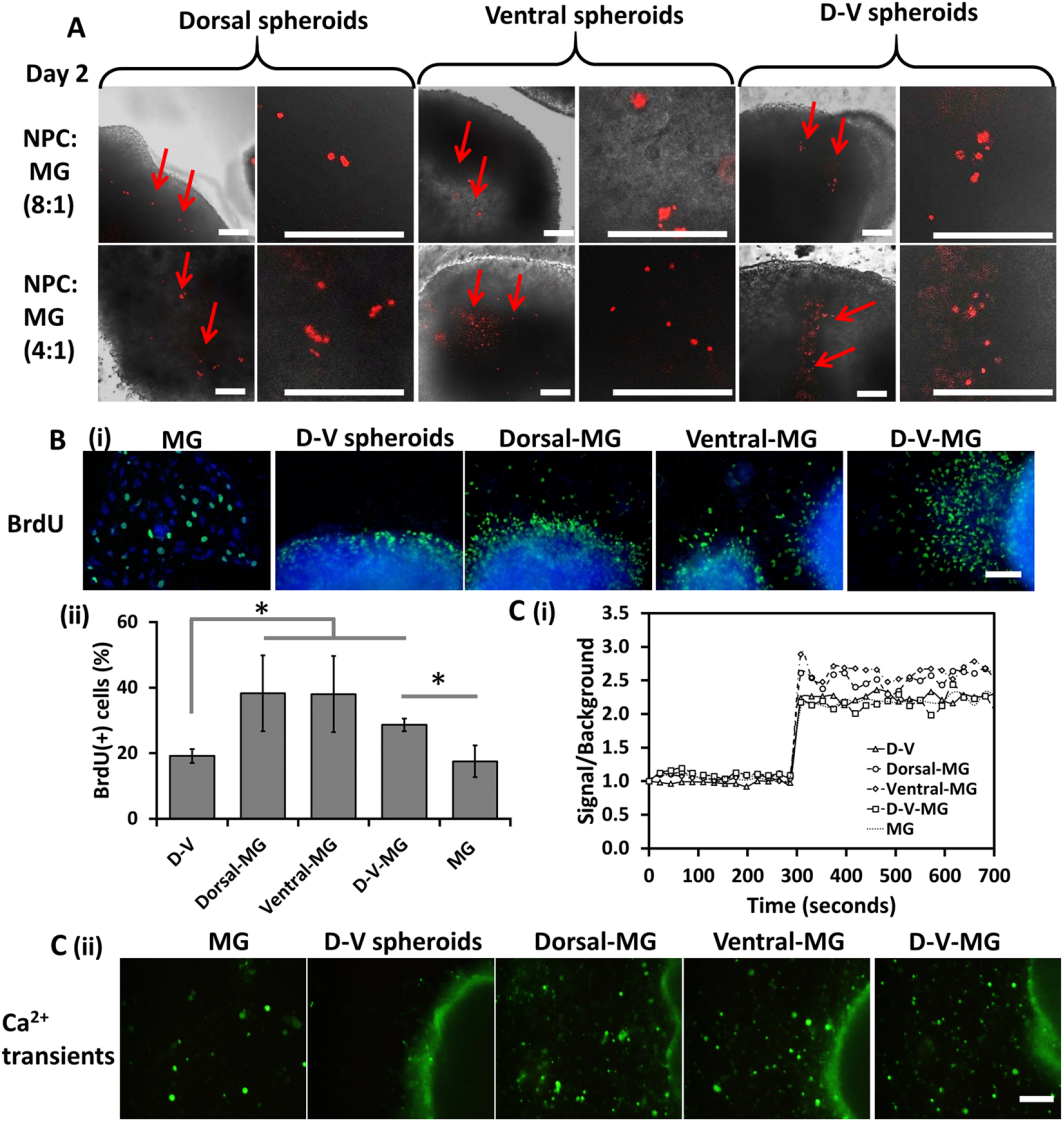
Co-Culture of microglia-like cells with ventral, dorsal or ventral/dorsal organoids: BrDU and Ca^2+^ transient assays. Spatial cell distribution of microglia-like cells in co-culture. Microglia-like cells were labeled with microdevices. (**A**) Overlay of phase contrast images with fluorescent images to show the integration of microglia-like cells within dorsal spheroids, ventral spheroids; and ventral/dorsal spheroids. Red arrows point to the microglia inside the spheroids. Scale bar: 100 μm. (**B**) BrdU assays for cell proliferation. (i) Fluorescent images of BrdU^+^ cells. Scale bar: 100 μm. (ii) Quantification of BrdU^+^ cells. (**C**) Ca^2+^ transient assays (induced by ADP). (i) Quantification of Ca^2+^ fluorescent signals; (ii) Fluorescent images of Ca^2+^ transient expression. Scale bar: 100 μm. *Indicates *p* < 0.05 for the different test conditions.

CellTracker Green or microdevice-labeled microglia-like cells migrated into the cortical spheroids after co-culture. MG incorporation increased over 2 days of culture (Fig. 6A and Supplementary Fig. S6). The NPC to MG ratio at 4:1 showed a high portion of microglia incorporation into the spheroids. Microglia incorporation was faster in the dorsal spheroids than the ventral spheroids, while the dorsal/ventral (D-V) spheroids showed the intermediate microglia mobility. BrdU incorporation (showing cells in S phase of cell cycle) was determined for the co-culture (Fig. 6Bi). There were 17.6–19.1% of BrdU^+^ cells for microglia only and D-V spheroids (Fig. 6Bii), while 38.3 ± 11.5%, 38.0 ± 11.6%, and 28.6 ± 1.9% of BrdU^+^ cells were observed for dorsal-MG, ventral-MG, and D-V-MG groups respectively. Histological sections of D-MG and V-MG spheroids showed the cellular distribution throughout the spheroids without necrotic center (Supplementary Fig. S7A). The microglia incorporation was confirmed by P2RY12 (red)/β-tubulin III (green) expression in the histological sections (Supplementary Fig. S7B).

Microglia-like cells respond to the ATP/ADP release through P2RY12/13 (purinergic receptors) at the damage area^33^, which would result in the intracellular Ca^2+^ transients^35^. ADP-evoked intracellular Ca^2+^ transients were observed in the derived MGs (Fig. 6Ci and Supplementary Video 1), which is the distinctly different characteristics compared to macrophages (i.e., negative ADP-Ca^2+^ transients for macrophages)^58^. The dorsal-MG (D-MG), ventral-MG (V-MG), and D-V-MG groups showed instant intracellular Ca^2+^ transients after ADP challenge (Fig. 6Ci and Supplementary Fig. S8). Cells from V-MG spheroids showed more ADP-responsive signals than the other groups (Fig. 6Cii).

**Figure 7:**
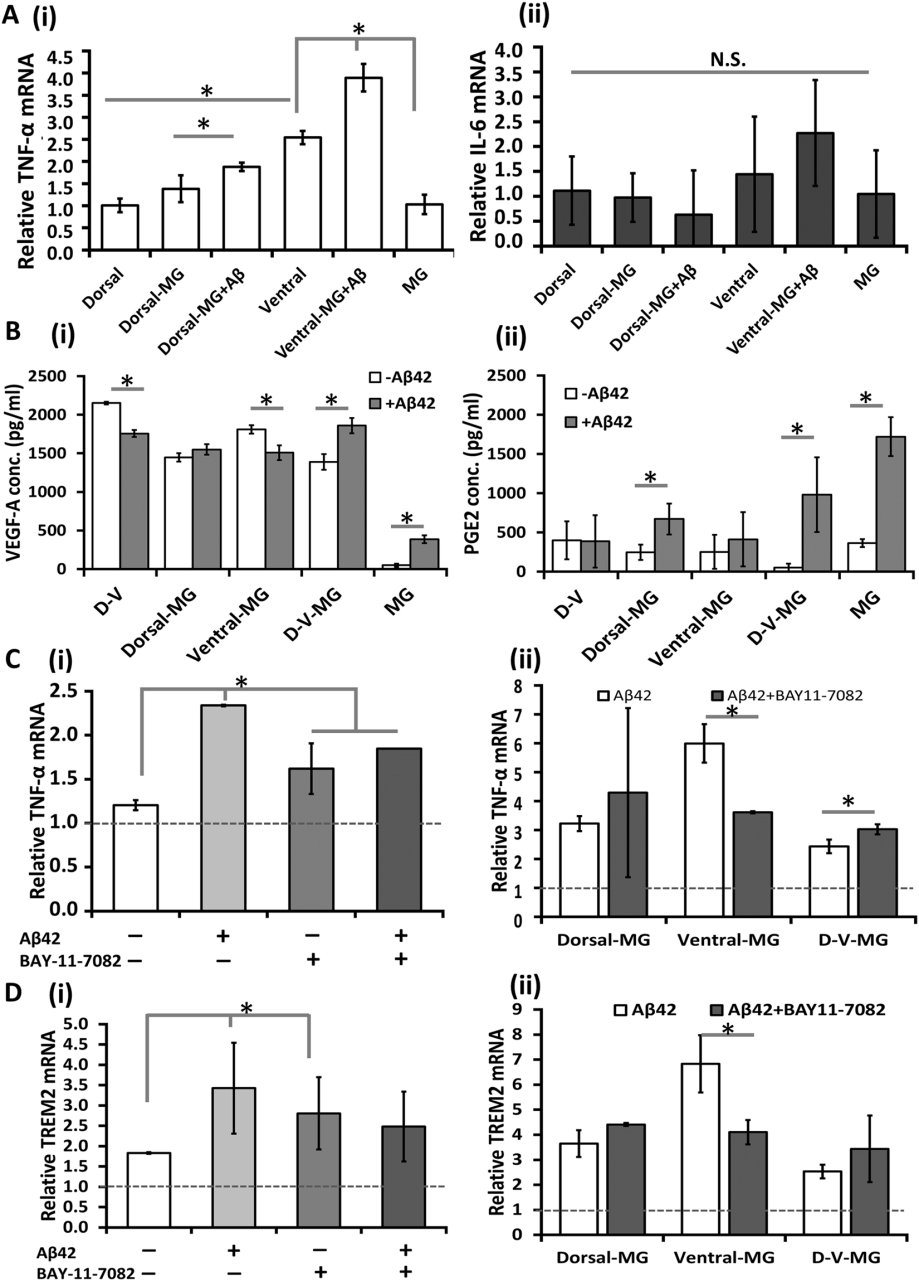
Co-Culture of microglia-like cells with ventral, dorsal or ventral/dorsal organoids: immunomodulatory properties. Co-cultured organoids (day 35) were stimulated with Aβ42 oligomers for 72 hours before the analysis. (**A**) RT-PCR analysis of (i) TNF-α and (ii) IL-6 gene expression (n = 3). (**B**) The secretion of cytokines (i) VEGF-A and (ii) PGE2 (n = 3) determined by ELISA assay. (**C**) Effect of NF-kB inhibitor BAY11–7082 (10 μM) on mRNA expression of TNF-α determined by RT-PCR (n = 3). (i) MGs and (ii) Co-cultured spheroids/organoids treated with BAY11-7082 in the presence of Aβ42 oligomers; Dash line represents the dorsal NPC only control. (**D**) Effect of NF-kB inhibitor BAY11-7082 (10 μM) on mRNA expression of TREM2 determined by RT-PCR (n = 3). (i) MGs and (ii) co-cultured spheroids/organoids treated with BAY11-7082 in the presence of Aβ42 oligomers. Dash line represents the dorsal NPC only control. *Indicates *p* < 0.05 for the different test conditions.

The immune response of co-cultured organoids to pro-inflammatory stimuli Aβ42 oligomers was examined (Fig. 7). Dorsal spheroids and microglia control showed comparable levels of TNF-α gene expression (Fig. 7A). D-MG group had similar TNF-α gene expression to dorsal group, while Aβ42 oligomer stimulation upregulated TNF-α expression for the D-MG and V-MG groups (Fig. 7A). No significant difference was observed for IL-6 expression. Co-culturing microglia with dorsal or ventral spheroids also showed differential secretion of VEGF-A and PGE2 (Fig. 7B). D-V-MG spheroids had a higher level of VEGF-A and D-MG spheroids had a higher level of PGE2 after Aβ42 oligomer stimulation, while V-MG spheroids showed insignificant PEG2 secretion and less VEGF-A expression after Aβ42 oligomer stimulation. The reactive oxygen species (ROS) production increased for MG and V-MG groups after Aβ42 oligomer treatment (Supplementary Fig. S9). But for D-MG and D-V-MG groups, the ROS increase was minimal. To further confirm the immune response, a NF-kB inhibitor BAY11-7082 was used to treat Aβ42 oligomer-stimulated MGs (Fig. 7Ci) and the co-cultured organoids (Figure Cii). BAY11-7082 treatment reduced TNF-α expression for the MG and V-MG groups. Similarly, TREM2 expression was increased when the cells were treated with Aβ42 oligomers (Fig. 7D). BAY11-7082 treatment reduced TREM2 expression especially for the V-MG group.

### Transcriptome analysis of MG and D-MG co-cultures

**Figure 8:**
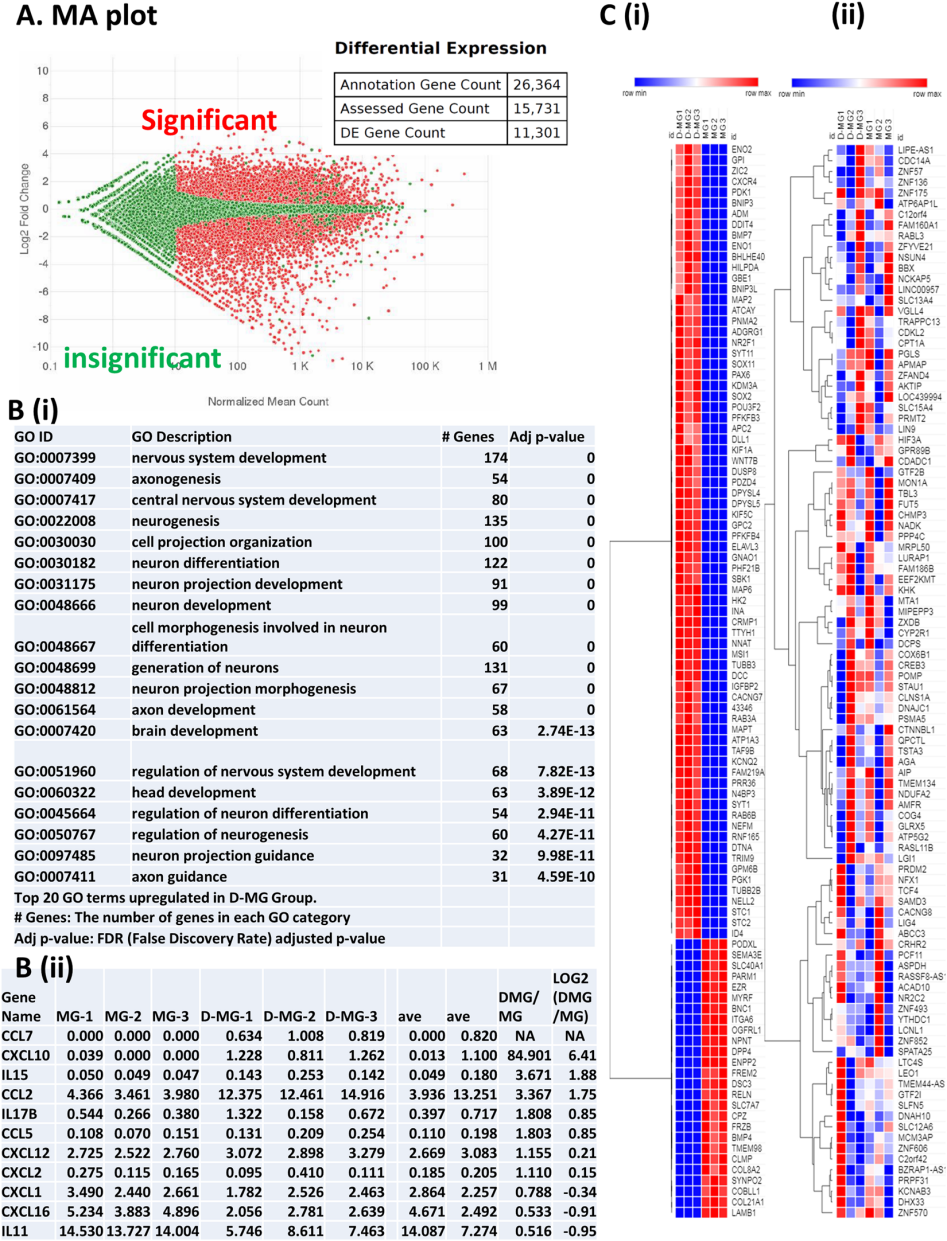
Genomics analysis for microglia-like cells and D-MG spheroids (day 33). (**A**) A MA-plot, in which the plot visualizes the differences between the pairwise comparison of the two sets of samples. The log transformed fold change for each gene is plotted along the y axis, and the mean read count value is plotted on the x axis. Genes with more numerous read counts appear further to the right on the plot. Red points are genes that are up or downregulated significantly (FDR-adjusted p-value < 0.05). (**B**) (i) Example of “GO” analysis for top 500 upregulated genes in Biological Process. (ii) A table of expressed genes associated with chemokines and cytokines. The FPKM (fragments per kilobase per million reads) normalized values for these genes are listed for both samples. The numbers are the Log2 values of ratios of D-MG to MG. Negative values indicate that the genes are present in higher amounts in MG group, while positive values indicate that the genes are present in higher amounts in D-MG group. (**C**) (i) Heat map of top 100 upregulated or downregulated genes between MG and D-MG groups. (ii) Heat map of top 100 commonly expressed genes (least different expression levels).

The RNA sequencing analysis (RNA-Seq) on the MG and the D-MG groups (4:1 ratio at day 33) was performed to further characterize MG alone and MG in cortical spheroids. For each group, three independent biological replicates were analyzed. An average of 15,731 genes of the 26,364 genes in the reference genome were detected. A pairwise comparison of the two groups using DESeq2 found 11,301 differentially expressed genes (Fig. 8A and Supplementary Spreadsheet 1). This is expected since the cultures represent different cell types and a majority of the cells in the D-MG group were neuronal. As evidence that the co-culture does contain microglia, 37 of microglia-specific genes, e.g., MERTK, GPR34, PROS1, GAS6, ITGAM (CD11b), CX3CR1, and TMEM119, were found in both groups (Table 1). ITGAM, GPR34, CX3CR1 etc. were enriched in D-MG group, while MERTK, TLR3, TMEM119, CD200R1, CD74 (HLA-DR), PROS1 etc. were highly expressed the MG group. Other immune functional genes such as CD163, CD14, RUNX1, AIF1 were similarly expressed in the two groups. The interferon pathway related genes were enriched in the MG group (Table 2). The SIGLEC family genes were differentially expressed in the two groups. Microglia heterogeneity was known by the expression of immunoreceptors containing activating and inhibitory members^40^. Our data show that the D-MG group exhibited higher levels of immunoreceptor genes in activating members, but the MG group contained higher levels for most of genes in inhibitory members (except SIGLEC5 and CD200) (Table 2).

**Table 1:** Microglia-related genes expressed in both MG and D-MG groups.

Gene Name	MG-1	MG-2	MG-3	D-MG-1	D-MG-2	D-MG-3	Ave (MG)	Ave (D-MG)	DMG/MG	LOG2 (DMG/MG)
SPI1	0.034	0.000	0.000	0.082	0.039	0.095	0.011	0.072	6.411	**2.68**
ITGAM	0.010	0.010	0.028	0.109	0.069	0.042	0.016	0.073	4.634	**2.21**
SIGLEC11	0.030	0.000	0.000	0.109	0.017	0.000	0.010	0.042	4.223	**2.08**
IL34	0.197	0.145	0.207	0.839	0.543	0.592	0.183	0.658	3.597	**1.85**
ENTPD1	0.401	0.346	0.404	1.300	1.278	1.235	0.384	1.271	3.310	**1.73**
GPR34	0.049	0.024	0.103	0.119	0.057	0.173	0.059	0.116	1.985	**0.99**
CX3CR1	0.039	0.025	0.018	0.063	0.045	0.055	0.027	0.054	1.979	**0.98**
TLR4	0.082	0.040	0.057	0.089	0.095	0.115	0.060	0.100	1.675	**0.74**
CD163	0.068	0.066	0.047	0.110	0.065	0.064	0.060	0.080	1.318	**0.40**
CD14	0.497	0.281	0.439	0.668	0.333	0.406	0.406	0.469	1.156	**0.21**
CSF1R	0.235	0.321	0.329	0.157	0.313	0.481	0.295	0.317	1.075	**0.10**
RUNX1	1.054	1.122	1.093	0.885	1.228	1.354	1.090	1.156	1.061	**0.08**
CNR1 (CB1)	3.040	2.760	3.118	2.950	2.483	2.992	2.973	2.808	0.945	**−0.08**
AIF1	0.137	0.139	0.128	0.142	0.123	0.079	0.135	0.115	0.853	**−0.23**
CTSD	181.030	141.431	181.199	126.489	165.501	133.915	167.887	141.968	0.846	**−0.24**
GAS6-AS1	0.106	0.310	0.148	0.192	0.122	0.075	0.188	0.130	0.692	**−0.53**
ITM2B	187.148	165.841	188.395	98.238	130.503	126.546	180.461	118.429	0.656	**−0.61**
IRF8	0.249	0.191	0.248	0.129	0.082	0.150	0.229	0.121	0.527	**−0.93**
GAS6	23.550	18.779	22.989	10.804	11.318	10.370	21.772	10.831	0.497	**−1.01**
SELPLG	1.070	0.597	0.926	0.227	0.511	0.503	0.864	0.414	0.479	**−1.06**
PTPRC	0.013	0.025	0.009	0.000	0.022	0.000	0.016	0.007	0.476	**−1.07**
MERTK	10.754	12.517	10.041	5.241	4.409	5.152	11.104	4.934	0.444	**−1.17**
ITGB5	26.159	21.157	26.867	9.465	8.988	12.065	24.728	10.172	0.411	**−1.28**
TMEM119	0.455	0.154	0.636	0.170	0.162	0.173	0.415	0.168	0.406	**−1.30**
CD200R1	0.296	0.231	0.221	0.072	0.160	0.056	0.249	0.096	0.385	**−1.38**
CD74 (HLA-DR)	20.273	16.700	19.614	5.158	6.417	8.057	18.862	6.544	0.347	**−1.53**
TLR2	0.250	0.258	0.214	0.101	0.016	0.118	0.241	0.078	0.326	**−1.62**
CD274	0.128	0.113	0.233	0.016	0.045	0.091	0.158	0.050	0.318	**−1.65**
CD68	21.240	19.006	23.817	6.311	7.493	6.363	21.354	6.722	0.315	**−1.67**
CST3	140.183	95.749	136.805	26.568	30.377	30.928	124.246	29.291	0.236	**−2.08**
PROS1	14.510	14.664	14.544	2.775	3.319	3.709	14.573	3.267	0.224	**−2.16**
SIRPA (CD172a)	5.045	4.001	4.854	0.885	0.880	1.207	4.634	0.991	0.214	**−2.23**
TLR3	0.295	0.243	0.282	0.057	0.018	0.088	0.274	0.054	0.198	**−2.34**
CSF1	14.002	12.015	13.027	2.243	2.451	2.905	13.015	2.533	0.195	**−2.36**
RGS10	27.176	23.513	24.422	3.360	3.991	5.138	25.037	4.163	0.166	**−2.59**
OLFML3	238.303	208.748	232.245	29.021	26.068	31.671	226.432	28.920	0.128	**−2.97**
TYROBP	2.639	2.656	2.124	0.194	0.371	0.226	2.473	0.264	0.107	**−3.23**
P2RY11	0.335	0.537	0.033	2.208	1.441	1.384	0.302	1.678	5.557	**2.47**
P2RY14	0.000	0.033	0.023	0.061	0.058	0.047	0.019	0.055	2.967	**1.57**
P2RY1	1.019	1.396	1.446	1.552	1.339	1.395	1.287	1.429	1.110	**0.15**
P2RY4	0.115	0.169	0.242	0.140	0.267	0.081	0.175	0.163	0.929	**−0.11**
P2RY2	0.296	0.238	0.333	0.116	0.282	0.276	0.289	0.225	0.777	**−0.36**
P2RY6	1.319	1.129	1.492	0.504	0.446	0.607	1.313	0.519	0.395	**−1.34**

The FPKM (fragments per kilobase per million reads) normalized values for these genes are listed for all samples. The numbers in the last column are the Log2 values of ratios of D-MG to MG. Negative values indicate that the genes are present in higher amounts in MG group, while positive values indicate that the genes are present in higher amounts in D-MG group.

The genes were separated into different groups based on: upregulated in DMGs (>+0.50 fold); similar expression (−0.49 to +0.49 fold); upregulated in MGs (−0.50 to −2.00 fold); highly upregulated in MGs (<−2.00 fold), and P2RY receptors.

**Table 2:** Microglia immunophenotype suggests differences in immune vigilance for D-MG and MG groups.

Gene Name	MG-1	MG-2	MG-3	D-MG-1	D-MG-2	D-MG-3	Ave (MG)	Ave (D-MG)	DMG/MG	LOG2 (DMG/MG)
**Interferon pathway**										
STAT1	18.863	18.003	17.906	12.591	16.296	13.932	18.258	14.273	0.782	−0.36
STAT4	0.321	0.448	0.235	0.242	0.177	0.259	0.335	0.226	0.675	−0.57
OAS1	0.238	0.232	0.199	0.087	0.055	0.101	0.223	0.081	0.362	−1.47
IFIT2	0.974	0.912	0.756	0.164	0.172	0.229	0.881	0.189	0.214	−2.22
PLSCR1	12.662	11.825	11.660	1.949	1.710	2.083	12.049	1.914	0.159	−2.65
IFITM3	465.310	367.927	441.155	53.291	57.131	73.014	424.797	61.145	0.144	−2.80
**SIGLEC family**										
SIGLEC1	0.028	0.021	0.020	0.111	0.114	0.129	0.023	0.118	5.178	2.37
SIGLEC11	0.030	0.000	0.000	0.109	0.017	0.000	0.010	0.042	4.223	2.08
SIGLEC14	0.000	0.022	0.000	0.000	0.052	0.032	0.007	0.028	3.817	1.93
SIGLEC5	0.000	0.018	0.000	0.023	0.043	0.000	0.006	0.022	3.615	1.85
SIGLEC16	0.029	0.029	0.082	0.143	0.170	0.166	0.047	0.160	3.415	1.77
SIGLEC15	0.094	0.092	0.088	0.114	0.073	0.044	0.091	0.077	0.845	−0.24
SIGLEC10	0.125	0.190	0.078	0.135	0.081	0.039	0.131	0.085	0.649	−0.62
SIGLEC6	0.197	0.600	1.104	0.133	0.203	0.279	0.634	0.205	0.324	−1.63
SIGLEC9	0.023	0.046	0.033	0.028	0.000	0.000	0.034	0.009	0.279	−1.84
**Activating members**										
TREM1	0.015	0.015	0.151	0.169	0.412	0.000	0.061	0.194	3.195	1.68
CD300LB	0.062	0.020	0.115	0.200	0.119	0.058	0.066	0.126	1.916	0.94
CD300LF	0.023	0.022	0.000	0.028	0.000	0.032	0.015	0.020	1.329	0.41
**Inhibitory members**										
SIGLEC5	0.000	0.018	0.000	0.023	0.043	0.000	0.006	0.022	3.615	1.85
CD200	11.187	13.126	11.137	18.303	20.706	20.599	11.817	19.869	1.681	0.75
CD300A	0.974	0.829	0.908	0.728	1.012	0.529	0.904	0.756	0.837	−0.26
CD47	12.758	15.348	12.051	9.623	9.966	9.872	13.386	9.820	0.734	−0.45
CD200R1	0.296	0.231	0.221	0.072	0.160	0.056	0.249	0.096	0.385	−1.38
SIRPA	5.045	4.001	4.854	0.885	0.880	1.207	4.634	0.991	0.214	−2.23
CD22	1.090	1.023	0.902	0.157	0.083	0.121	1.005	0.120	0.120	−3.06

The FPKM (fragments per kilobase per million reads) normalized values for these genes are listed for all samples. The numbers in the last column are the Log2 values of ratios of D-MG to MG. Negative values indicate that the genes are present in higher amounts in MG group, while positive values indicate that the genes are present in higher amounts in D-MG group.

To gain insight into the function of the differentially expressed genes, gene ontology (GO) and KEGG pathway enrichment were examined for the top 500 genes that were upregulated in each group for categories of Biological Process, Cellular Component, and Molecular Function (Supplementary Spreadsheet 2). The examples of “GO” analysis regarding neuron development, morphogenesis, and neural protection were shown in Fig. 8Bi. The expression of chemokines such as CXCL1, 2, 12, 16, and CCL2, 5 was also observed in both groups, but CCL7 and CXCL10 were much higher in the D-MG group (Fig. 8Bii). From the heatmap of top 100 upregulated or downregulated genes (Fig. 8Ci), the MG cells were enriched with collagens and laminin related genes (e.g., COL8A2, COL21A1, LAMB1, ITGA6, and RELN), while the cells of D-MG group were enriched with Glypican 2 (GPC2), CXCR4, Dll1 (a Notch ligand for cell-cell communication), and STC1 (a glycoprotein hormone involved in calcium/phosphate homeostasis). MG group also enriched FRZB, a Wnt-binding protein, and MYRF, the myelin regulatory factor. The D-MG group enriched the genes related to synaptic function, SYT1 and SYT11, and neuroprotective gene ENO2.

A list of 100 genes that share similar expression levels in both cultures was examined (Fig. 8Cii). From “GO” analysis, these genes are implicated in general cellular functions, such as basal transcriptional machinery binding, RNA polymerase II core binding, and several transmembrane transporter activities (Supplementary Table S3) The expression of genes associated with Alzheimer’s disease such as APOE, PSEN1, PSEN2, PICALM, and APP were expressed in both groups (Supplementary Table S4). But MAPT, MEF2C, CASS4 were higher in the D-MG group while APOE and PTK2B were higher in the MG group. For genes-related to cortical neurons, they were all higher in the D-MG group as expected (Supplementary Table S5). In addition, oligodendrocyte-associated genes (e.g., Olig1, Olig2, ASCL1) were highly expressed in the D-MG groups (Supplementary Table S6), as well as astrocyte-associated genes (e.g. SLC1A2, S100B) (Supplementary Table S7) and genes related to brain-specific pericytes (e.g., ZIC1 and MCAM, the encoding gene for CD146) (Supplementary Table S8)^59^.

## Discussions

### Importance for studying hiPSC-microglia

Most of current microglia studies, in particular under disease condition, have to use mouse cells due to the limited access to human microglia^43,60^. iPSCs provide a promising platform to generate human microglia with the patients’ genetic backgrounds. There are three microglia groups in human brain: homeostatic, intermediate, and disease-associated microglia (DAM)^43,61^. A shift in microglial phenotype, increased cytokine production, and reduced phagocytic capacity were observed during neural degeneration^62^. This DAM was found to be activated with a two-step mechanism from homeostatic microglia: (1) triggering receptor expressed on myeloid cells 2 (TREM2)-independent mechanism to intermediate microglia; the reduction in phenotypic markers CX3CR1 and P2RY12 and the upregulation of APOE were observed^43^. (2) TREM2-dependent activation, showing the upregulation of phagocytic and lipid metabolism genes^34,43^. TREM2 deficiency promotes microglia cell death and inhibits Wnt/β-catenin pathway^63^. During neural degeneration, microglia should clear the Aβ fibrils and secrete neuro-inflammatory cytokines^34,42^, but prolonged activation of microglia is detrimental to brain function. All these studies underscore the importance of investigating the phenotype and function of microglia in healthy or diseased brain environment.

### Generation and characterization of hiPSC-microglia

Generation of hPSC-derived microglia-like cells can be differentiated through a CD235a^+^ intermediate state or through a monocyte/macrophage intermediate^30^. Differentiation microglia-like cells from hPSCs was firstly reported in 2016^33^. Embryoid body formation in suspension using CSF1 and IL-34 was performed to obtain progenitors expressing VE-cadherin, c-kit, CD41 and CD235a, the markers of early yolk sac myelogenesis. Further differentiation generated CD11b^+^IBA-1^+^ semi-adherent cells with vacuolated and round morphology (about 8 weeks). In 2017, a two-step protocol was developed to generate microglia cells from hiPSCs in 5 weeks^34^. Primitive hematopoietic progenitor cells (CD41^+^CD43^+^CD235a^+^) were obtained from iPSCs using Activin A, BMP-4, FGF2 and LiCl, followed by FGF2, VEGF, TPO, SCF, IL-3 and IL-6. Similarly, a monolayer-based differentiation used Activin A, BMP-4, SCF and VEGF for the generation of CD34^+^CD43^+^ cells^37^, or with BMP-4 followed by FGF-2, SCF and VEGF^35^. Compared to previous protocols^37^, our study used the monolayer-based simplified protocol (i.e., remove hypoxia and feeder culture) through mesoderm intermediate to generate microglia-like cells with proper phenotype: 70 ± 6% CD11b and 80 ± 5% IBA-1 at Day 28; 68 ± 12% P2RY12 and 51 ± 10% CX3CR1 at Day 33. The role of VEGF during the development was elucidated: in the absence of VEGF, the KDR at day 5–7 and CX3CR1 at day 30–33 were lower. Functional analysis showed the phagocytosis ability, the increased TNF-α expression and cytokine secretion to pro-inflammatory Aβ42 oligomers, which were reported to modulate microglia responses by TREM2 binding^64^. In addition, ADP-evoked intracellular Ca^2+^ transients were observed in iPSC-MGs, a characteristic function distinctly different from macrophages.

### Co-culture of microglia with brain-region dependent spheroids

To investigate how central nervous system microenvironments affect microglia maturation and homeostasis, microglial cells were co-cultured with rat hippocampal neurons^34^ or with isogenic cortical neurons to acquire microglia characteristics in 2D cultures^36^. The co-culture upregulated genes in neural protection and suppressed pro-inflammatory signaling, maintaining a homeostatic state^65^. Microglia cells were co-cultured with 3D whole brain organoids in only one study showing that microglia can mature, ramify, and respond to injury; another study derived microglia within cerebral organoids^34,51^. The novelty of our study is to investigate the neural-microglia interactions for brain “region-specific” organoids (dorsal or ventral). The dorsal or ventral identity was confirmed by the expression of TBR1/PAX6 or NKX2.1/PROX1 respectively, and displayed synaptic activities and action potentials.

Our co-culture results indicate that the isogenic microglia-like cells showed differential migration ability and immune response with dorsal or ventral cortical organoids. The immune responses of co-cultured organoids to pro-inflammatory stimuli showed higher TNF-α expression for ventral-MG co-culture. The co-culture also stimulated cell proliferation (higher BrdU^+^ cells), reduced ROS expression, and exhibited the response to ADP treatment for intracellular Ca^2+^ transients. During neural degeneration, knockdown of the low-density lipoprotein receptor-related protein (LRP1), a receptor of Aβ peptides, leads to activation of c-Jun N-terminal kinase and NF-kB pathways and enhanced the sensitivity to LPS response^60^. Similarly, in our study, NF-kB pathway is involved in Aβ42-induced pro-inflammatory response (through TREM2 binding). The BAY11-7082 treatment blocked the NF-κB signaling and reduced TNF-α expression in the activated microglia-like cells, especially when co-culturing with ventral organoids.

Our genomics analysis showed the differential expression of microglia-related genes (Table 1) and genes related to neural degeneration (similar to Haenseler *et al*., 2017 with 2D co-culture^36^). Distinct differences between microglia-like cells and co-cultured cells with 3D forebrain spheroids were observed, in particular, for immunoreceptors containing activating and inhibitory members (Table 2)^40^, which indicates that the immunophenotype has heterogeneity for the MG and the D-MG groups. The genes related to ECMs (Fig. 8 and Supplementary Spreadsheet 3) were also differentially expressed. The microglia-like cells were enriched with collagens and laminin related genes, while the co-cultured cells expressed higher levels of Glypican 2 and Notch ligand. The co-cultured cells had higher levels of genes related to neurogenesis, synaptic function, as well as neuroprotection. ECMs and cytokines are important to activate microglia function^66^, thus the neural microenvironment provided by cortical spheroids should promote microglia function. To date, the transcriptome analysis of hiPSC-derived microglia showed the similarity to human fetal microglia^35,37,39^. However, the brain region-dependent diversity of microglia phenotype and the response to neural degeneration^40,43^ may require microenvironment provided by brain region-specific spheroids. Therefore, our transcriptome analysis focus on the comparison of the presence and absence of 3D cortical microenvironment.

3D spheroids experiences hypoxia and glucose starvation compared to 2D cultures^67^. Metabolic reprogramming may occur from oxidative phosphorylation to aerobic glycolysis in 3D spheroids^68^, which favors microglia M1 polarization^69^. Activation of mTOR (PIK3/AKT/mTOR pathway) and p53, repression of canonical Wnt and NF-kB pathway activities, and higher ATP levels could be observed in 3D spheroid culture^67^, as well as our genomics analysis summarized in a separate manuscript^68^. So the co-cultured 3D organoids, as shown in this study, more resemble physiological brain tissue environment than 2D neural cultures.

### Implication for disease modeling

Patient-derived 3D brain organoids from hiPSCs have been widely investigated recently as a powerful platform for drug screening for neurodegenerative disease. As the only resident macrophages and indispensable cellular component of blood-brain barrier (BBB), most recent brain organoids currently lack the microglia. A couple of recent studies demonstrated the importance of adding microglia into the neural constructs by observing up-regulated genes related to immune functions, such as IBA-1, CD14, and TREM2, compared to those without microglia^17,70^. However, the microglia-functionalized brain organoids in a diseased environment have not been well investigated. Our lab recently reported the derivation of AD-patient derived cortical organoids to recapitulate neurodegenerative microenvironment and investigate its response to potential drug treatment^46^. AD-related microenvironment including a higher level of Aβ42, elevated pro-inflammatory gene expression, and altered matrix remodeling proteins were recapitulated in the cortical organoids derived from AD-associated hiPSCs. This study performed the functional characterization of microglia within different regions of forebrain organoids. The long-term goal is to fabricate next-generation brain organoids with additional cellular components (e.g., microglia, BBB properties, astrocytes, and oligodendrocytes) from hiPSCs for disease modeling, drug screening, and possibly cell therapy^71^.

## Conclusions

In summary, the microglia-like cells were derived from hiPSCs and integrated with brain region-specific organoids. Co-culturing isogenic microglia-like cells with hiPSC-derived dorsal and ventral organoids showed differential migration ability, Ca^2+^ transients imaging, and the response to pro-inflammatory stimuli. D-MG group showed higher anti-inflammatory cytokine secretion, while V-MG group showed higher TNF-α expression under Aβ42 stimulation. The co-culture (especially V-MG group) also stimulated cell proliferation (higher BrdU^+^ cells) and reduced ROS expression, better resembling tissue-specific microenvironment. Transcriptome analysis exhibited microglia-related genes that were differentially expressed in MG and D-MG groups. This study should advance our understanding of the effects of microglia on brain tissue function towards engineering complex next generation of brain organoids.

## Methods

### Undifferentiated hiPSC culture

Human iPSK3 cells were derived from human foreskin fibroblasts transfected with plasmid DNA encoding reprogramming factors OCT4, NANOG, SOX2 and LIN28 (kindly provided by Dr. Stephen Duncan, Medical College of Wisconsin, and Dr. David Gilbert, Department of Biological Sciences of Florida State University)^72,73^. Human iPSK3 cells were maintained in mTeSR serum-free medium (StemCell Technologies, Inc., Vancouver, Canada) on 6-well plates coated with growth factor reduced Geltrex (Life Technologies). The cells were passaged by Accutase dissociation every 5–6 days and seeded at 1 × 10^6^ cells per well of 6-well plate in the presence of 10 μM Y27632 (Sigma) for the first 24 hours^46,74,75^.

### Microglia-like cell differentiation from hiPSCs

Microglia-like cells was induced from human iPSK3 cells in mTeSR serum-free medium on 24-well plate for two days. The medium was changed to fresh mTeSR1 on day −1. On day 0, cells were induced with 30 ng/mL VEGF (Peprotech), 30 ng/mL BMP4 (Peprotech), 40 ng/mL SCF (Peprotech), and 50 ng/mL Activin A (Invitrogen) in RPMI medium (Life Technologies) plus 2% B27. Four days later, the medium was changed with RPMI plus 2% B27 supplemented with 50 ng/mL SCF, 50 ng/mL Flt3L (Peprotech), 10 ng/mL IL-3 (Peprotech), 50 ng/mL GM-CSF (Peprotech), and 25 ng/mL BMP-4. On day 7 and day 10, half of old media were replaced with fresh media and the cells were grown for another 3–5 days. Cells were harvested on day 15 to analyze for hematopoietic markers using 1 mL of Accutase (Life Technologies) per well. Cells from one well of a 24-wp were split and replated into two wells of low-attachment 24-wp in DMEM, 10% defined fetal bovine serum (FBS), 1% Penicillin/Streptomycin (all from Life technologies), and 20 ng/mL of GM-CSF. After 2–5 days of myeloid progenitor expansion, non-adherent cells from one well of low attachment 24-wp were plated onto one well of tissue culture treated 24-wp in DMEM medium supplemented with 10% FBS, 1% Penicillin/Streptomycin, 20 ng/mL of IL-3, and 20 ng/mL of GM-CSF. The hematopoietic progenitors were further incubated at 37 °C and 5% CO_2_ for 1–2 weeks for differentiation to microglia.

### Functional analysis of microglia-like cells

Microglia-like cells (day 30–35) were stimulated in DMEM/10% FBS media containing Aβ42 oligomers (0.5 µM, Bachem)^45^, lipopolysaccharides (LPS) (100 ng/ml, Sigma), and dexamethasone (1 µM, Sigma). To prepare oligomers of Aβ42 peptides, biotinylated Aβ (1–42) (Bachem) was fully dissolved at 0.5 mg/mL in hexafluor-2-propanole (HFIP, Sigma). The solution was dispensed at 10 μL into a siliconized Snap-Cap microtube, put in a desiccator to evaporate HFIP, and thereafter stored at −80 °C. Oligomer solutions were prepared freshly for each experiment. The stock was dissolved in 10 µL of DMSO (to 105 μM) and incubated for 3 h at room temperature. The microglia-like cells that were not stimulated served as control. After 72 hours of stimulation, the cytokine secretion, including transforming growth factor (TGF)-β1, Prostaglandin E2 (PGE2), and vascular endothelial growth factor (VEGF), in the spent medium was analyzed by ELISA. TNF-a and IL-6 gene expression was analyzed by RT-PCR.

### Phagocytosis assay

Approximately 2 × 10^5^ microglia-like cells (day 30), hiPSC-derived neural progenitor cells (iNPCs) (day 21) and hiPSC-derived endothelial cells (iECs) (day 21)^76^ were replated onto 24-well plate in DMEM plus 10% FBS. The adherent cells were incubated with 1 ml fresh DMEM plus 10% FBS containing 0.25 × 10^8^ fluorescent (0.86 μm, flash red, 660/690 nm) micron-sized particles of iron oxide (MPIO)/mL (Bangs Laboratories, Fishers, IN, USA, Part number ME03F/9772) corresponding to the concentration at 2.5 μg Fe/mL^77,78^. The attached microglia-like cells that were not labeled with MPIO served as control. After 24-hour incubation, the cultures labeled with MPIO were extensively washed (10 times) with phosphate buffer saline (PBS). The cultures were then harvested for flow cytometry to quantify the cells with MPIO.

### Dorsal and ventral spheroid/organoid differentiation from hiPSCs

Undifferentiated iPSK3 cells (2 × 10^5^ cells) were seeded into low attachment 24-well plates in neural differentiation medium composed of DMEM/F-12 plus 2% B27. Y27632 (10 µM) was added during the seeding and removed after 24 h. For dorsal differentiation, the aggregates were treated with 10 µM SB431542 (Sigma) and 100 nM LDN193189 (Sigma) for 7 days. Then the spheroids were treated with FGF2 at 25 ng/mL for another 7 days^46,47,49^. To generate a ventral identity, the aggregates were treated with 10 µM SB431542 (Sigma), 100 nM LDN193189 (Sigma), and 5 µM IWP4 (Sigma) for 7 days. Then the spheroids were incubated with 5 µM IWP4 and 1 µM purmorphamine (Sigma) for another 7 days. For maturation, the spheroids were maintained in neural differentiation medium without growth factors for additional 14–38 days (total up to 52 days). The dorsal and ventral identity was characterized using histology, flow cytometry, and RT-PCR.

### Preparation of microdevices for labeling microglia-like cells

#### Materials

Poly(allylamine hydrochloride) (PAH) was purchased from Beantown chemical (molecular weight: 120,000–200,000). Poly(sodium-p-styrenesulfonate) (PSS) was purchased from Acros Organics (average molecular weight: 70,000). Poly(n-propyl methacrylate) (PPMA) was purchased from Scientific Polymer Products, Inc (average molecular weight: 150,000). Gelatin (from porcine skin, gel strength ~300 g Bloom) was purchased from Sigma-Aldrich. Octadecyl rhodamine B chloride (R18) was purchased from Biotium, Inc.

#### Preparation of gelatin-coated glass slides

A 60 °C gelatin solution (10 wt% in water) was dip-coated onto a glass slide. The solution was dried in air at room temperature for 12 hours.

#### Preparation of micro contact printing (µCP) stamps

Monomer and curing agent of Sylgard 184 PDMS kit (Dow Corning) were mixed at 10:1 weight ratio. The mixture was degassed and poured on a silicon master with micro-patterns prepared by photolithography. The mixture was then cured at 37 °C for 24 hours to form a PDMS slab. The PDMS slab was peeled off from the master and cut into small stamps. The stamp had an array of circular pillars with a diameter of 5 µm and a center-to-center distance of 10 µm in the square lattice.

#### Fabricating PAH/PSS/PAH/PPMA-R18 particles

Three layers of polyelectrolyte (PAH/PSS/PAH) were deposited onto the PDMS stamp via layer-by-layer technique. The stamp was soaked in polyelectrolyte solution for 15 min to deposit the specific layer. Polyelectrolyte solution used for the first layer: PAH solution (1 wt% in water, pH = 0, containing 150 mM NaCl); second layer: PSS solution (1 wt% in water); third layer: PAH solution (1 wt% in water, pH = 4, without NaCl). The stamp was washed with water and dried under a stream of nitrogen after soaking each layer. Then, an acetone solution of PPMA (5 wt%, containing 50 µg/mL R18) was spin-coated onto the PDMS stamp at 3000 rpm for 45 s. The stamp was brought into contact with a gelatin-coated glass slide which was placed on a hot plate at 100 °C. After 5 seconds, the stamp was peeled off, the particles were transferred onto the gelatin film.

#### Labeling microglial-like cell with particles

A PDMS chamber was placed onto a gelatin-coated glass slide which has the printed micro-arrays, forming a well whose bottom was covered by micro particles (each well has 7.85 × 10^5^ particles on the bottom). The well was placed in 6 °C refrigerator for 30 min. Then 1 × 10^5^ microglial cells (in 460 µL of phosphate-buffered saline, at 6 °C) was added into the well and kept at 6 °C for 30 min. The microglial cells precipitated and attached onto the particles. After 30 min, the well was brought into incubator (37 °C, 5% CO_2_) and incubated for 2 hours. The gelatin film was dissolved so the particles released, forming microglial/particle complexes during the process.

### Co-culture of dorsal or ventral spheroids/organoids with microglia-like cells (MGs)

Ventral, dorsal, or dorsal/ventral (D-V) spheroids were co-cultured with MGs at 4:1 ratio (8 × 10^5^ neurons to 2 × 10^5^ MGs) or 8:1 ratio (8:1 × 10^5^) in 50% DMEM/10% FBS and 50% neural differentiation medium composed of DMEM/F12 plus 2% B27. D-V spheroids were constructed by mixing ventral and dorsal spheroids (day 30–35) at 1:1 ratio for 24 hours. The migration and integration of CellTracker Green labeled MGs or cells labeled with microdevices within the neural spheroids were observed after 3 days of co-culture. The hybrid spheroids were harvested for RT-PCR analysis or for other characterizations.

Day 3 co-cultured D-V spheroids, D-V-MG, ventral-MG, dorsal-MG, and MG monoculture were treated with 1 µM Aβ42 oligomers for 3 days in the presence or absence of 10 µM BAY11-7082 (Sigma-Aldrich), a nuclear factor kB (NF-kB) signaling inhibitor. MG alone and MG pretreated with BAY11-7082 were used as controls. The RT-PCR analysis was performed for TNF-α and TREM2 expression.

### Enzyme-linked immunosorbent assay (ELISA) assay

To quantify the growth factors secreted by different spheroids, culture supernatants were collected at day 3 after stimulation. Concentrations of PGE2, VEGF-A, and TGF-β1 were measured by ELISA according to the manufacturers’ instructions (R&D Systems, Minneapolis, MN for PGE2; Life Technologies for TGF-β1 and VEGF-A).

### Immunocytochemistry

Briefly, the samples were fixed with 4% paraformaldehyde (PFA) and permeabilized with 0.2–0.5% Triton X-100. The samples were then blocked for 30 min and incubated with various mouse or rabbit primary antibodies (Supplementary Table S3) for four hours. For surface markers, no permeabilization was performed. After washing, the cells were incubated with the corresponding secondary antibody: Alexa Fluor® 488 goat anti-Mouse IgG_1_, 488 or 594 goat anti-Rabbit IgG, or 594 donkey anti-Goat IgG (Life Technologies) for one hour. The samples were counterstained with Hoechst 33342 and visualized using a fluorescent microscope (Olympus IX70, Melville, NY). For 5-Bromo-2′-deoxyuridine (BrdU) assay, the cells were incubated in medium containing 10 µM BrdU (Sigma) for four hours. The cells were then fixed with 70% cold ethanol and denatured using 2N HCl/0.5% Triton X-100 for 30 min in the dark. The samples were reduced with 1 mg/mL sodium borohydride for 5 min and incubated with mouse anti-BrdU (1:100, Life Technologies) in blocking buffer (0.5% Tween 20/1% bovine serum albumin in PBS), followed by Alexa Fluor® 488 goat anti-Mouse IgG_1_. The cells were counterstained with Hoechst 33342 and analyzed by a fluorescent microscope and the ImageJ software.

### Flow cytometry

To quantify the levels of various neural marker expression, the cells were harvested by trypsinization and analyzed by flow cytometry. Briefly, 1 × 10^6^ cells per sample were fixed with 4% PFA and washed with staining buffer (2% FBS in PBS). The cells were permeabilized with 100% cold methanol, blocked, and then incubated with primary antibodies against NKX2.1, SATB2, KDR, CD45, CD31, CD11b, IBA-1, CX3CR1, and P2RY12 (Supplementary Table S1) followed by the corresponding secondary antibody Alexa Fluor® 488 goat anti-Mouse IgG_1_, or 594 goat anti-rabbit IgG, or 594 donkey anti-goat IgG. For surface markers, no permeabilization was performed. The cells were acquired with BD FACSCanto™ II flow cytometer (Becton Dickinson) and analyzed against isotype controls using FlowJo software.

### Whole-patch clamping for electrophysiology

Whole-cell patch clamp was used to record from mature iPSK3-derived dorsal and ventral spheroids cultured on glass covered slips. Cover slips were washed three times with extracellular recording solution containing (in mM) 136 NaCl, 4 KCl, 2 MgCl, 10 HEPES, and 1 EGTA (312 mOsm, pH 7.39) and were incubated in this solution at room temperature during recording. Glass electrodes (resistance 1–5 MΩ) were filled with intracellular solution containing 130 mM KCl, 10 mM HEPES, and 5 mM EGTA (292 mOsm, pH 7.20). Cells were visualized under phase contrast with a Nikon Eclipse Ti-U inverted microscope and attached DS-Qi1 monochrome digital camera. Recordings were made with an Axopatch 200B amplifier (Molecular Devices) and digitized with a Digidata 1440A system (Molecular Devices). Ionic currents were recorded under a voltage clamp protocol (−60 mV to 135 mV in 15 mV steps, 250 ms in duration). Action potentials were recorded under a current clamp protocol (−100 pA to 200 pA in 20 pA steps, 800 ms in duration). Spontaneous post-synaptic currents were recorded under continuous voltage clamp at −80 mV for 2 min. Signals were filtered at 1 kHz and sampled at 10 kHz. Data was collected and analyzed using pCLAMP 10 software (Molecular Devices).

### Histology

For histology, the dorsal, ventral, or the D-V spheroids/organoids containing microglia-like cells were fixed in 10% formalin, dehydrated, and embedded in paraffin wax. The sections of 10 μm were cut, de-paraffinized, and stained with Lerner-2 Hematoxylin (Lerner Laboratories, Pittsburgh, PA) and Eosin-Y w/Phloxine (Richard-Allan Scientific, Kalamazoo, MI)^79^. The sections were also stained with various neural markers, including anti-SATB2, TBR1, BRN2, NKX2.1, PAX6, Glutamate, vGAT, β-tubulin III (Supplementary Table S1), to show different neural cell distribution and localization. Images were captured with an Olympus IX70 microscope with MagnaFire SP 2.1B software.

### Reverse transcription polymerase chain reaction (RT-PCR) analysis

Total RNA was isolated from different cell samples using the RNeasy Mini Kit (Qiagen, Valencia, CA) according to the manufacturer’s protocol followed by the treatment of DNA-Free RNA Kit (Zymo, Irvine, CA)^80^. Reverse transcription was carried out using 2 μg of total RNA, anchored oligo-dT primers (Operon, Huntsville, AL), and Superscript III (Invitrogen, Carlsbad, CA) (according to the protocol of the manufacturer). Primers specific for target genes (Supplementary Table S2) were designed using the software Oligo Explorer 1.2 (Genelink, Hawthorne, NY). The gene β-actin was used as an endogenous control for normalization of expression levels. Real-time RT-PCR reactions were performed on an ABI7500 instrument (Applied Biosystems, Foster City, CA), using SYBR1 Green PCR Master Mix (Applied Biosystems). The amplification reactions were performed as follows: 2 min at 50 °C, 10 min at 95 °C, and 40 cycles of 95 °C for 15 sec and 55 °C for 30 sec, and 68 °C for 30 sec. Fold variation in gene expression was quantified by means of the comparative Ct method: ${2}^{-({\rm{\Delta }}{C}_{ttreatment}-{\rm{\Delta }}{C}_{tcontrol})}$, which is based on the comparison of expression of the target gene (normalized to the endogenous control β-actin) between the compared samples.

### Reactive oxygen species (ROS) assay

ROS detection was performed using Image-iT^™^ Live Green Reactive Oxygen Species Detection kit (Molecular probes). Briefly, the spheroids and single cells were washed in Hank’s Balanced Salt Solution (HBSS), and incubated in a solution of 25 µM carbioxy-H_2_DCFDA for 30 min at 37 °C. The samples (+/−Aβ42 oligomers stimulation) were then washed and analyzed under fluorescence microscope or by flow cytometry. As positive control, the cells were incubated in a 100 µM tert-butyl hydroperoxide solution, prior to staining with carbioxy-H_2_DCFDA.

### Intracellular Ca^2+^ signaling assay

For calcium signaling, the samples were related on 1% Geltrex-coated 96-well plate and grown overnight. The growth medium was removed in each well and 100 μL of 1X Fluo-4 dye (Life Technologies) in assay buffer containing 1X HBSS and 20 mM HEPES (with 2.5 mM probenecid) was added into the wells and incubated at 37 °C for 30 min. The incubation was switched to room temperature for an additional 30 min. Baseline Ca^2+^ signals (I_494_/I_516_) were measured for more than 100 s, and then the calcium dye medium was replaced with 100 μL of 10 μM adenosine 5′-diphosphate (ADP, Sigma) solution in assay buffer (without probenecid). Ca^2+^ recordings were read on a fluorescent plate reader (FLX800, Bioinstrument Inc., Winooski, VT) using instrument settings appropriate for excitation at 494 nm and emission at 516 nm.

### RNA extraction and RNA-Seq cDNA Library Preparation

RNA was extracted from microglia-like cells only and D-MG group (4:1 ratio) at day 33 using the miRNeasy minikit (Qiagen). mRNA was isolated from the total RNA using an NEBNext Poly(A) mRNA Magnetic Isolation Module (New England Biolabs). cDNA libraries were generated from the isolated mRNA using an NEBNext Ultra RNA library prep kit for Illumina (New England Biolabs) and a unique 6 nucleotide index primer (NEBNext multiplex oligos for Illumina) was incorporated into each sample. The library construction was done according to the NEB manuals, modified for use with a Beckman Biomek 4000 at the Florida State University Biological Sciences core lab. The unique index (barcode) was added to each library to multiplex the six libraries in one lane of the sequencing run. The multiplexed sample was quantified with qPCR (Kapa Biosystems) specific for Illumina sequencing primers and the average fragment size was determined with a Bioanalyzer high sensitivity DNA chip (Agilent Technologies). 12 pM of the pooled sample was sequenced, with single end, 100 base reads on an Illumina HiSeq2500 located in the Translational Science Laboratory at the College of Medicine, Florida State University. The pooled data were demultiplexed into individual sample data and adapter primer sequences were removed^81^.

### RNA-Seq data analysis

Initial quality control analysis of each sequenced library was performed using fastQC software (http://www.bioinformatics.babraham.ac.uk/projects/fastqc). The sequencing reads were further analyzed using RNA-Seq Alignment version 1.1.1 (Illumina BaseSpace application). The reads were aligned with Tophat 2^82^ to the human genome (genome release GRCh38) using default parameters and counts for each gene were generated. This workflow uses Cufflinks to generate FPKM (fragments per kilobase per million reads) normalized values^83^. These normalized values account for differences in sequencing depth and the length of the gene. FPKM values were used to generate the heatmaps using Morpheus (Broad Institute; https://clue.io/morpheus). DESeq2 was used to determine statistically significant differentially expressed genes (a False Discovery Rate, FDR, of <0.05 was used). 15,585 genes were considered to be expressed in this study by the DESeq2 software due to low counts^84^. The top 500 genes that were upregulated and downregulated (1000 total genes) in the microglia culture versus the D-MG group were further assessed for GO, KEGG pathway and phenotype pathway analysis using Webgestalt^85,86^. The set of genes considered expressed in our dataset was used as the reference set to obtain significantly enriched pathways. Significant enrichment was determined in Webgestalt using the hypergeometric test and the Benjamini-Hochberg FDR method^87^ for multiple testing adjustment.

### Statistical analysis

Each experiment was carried out at least three times. The representative experiments were presented and the results were expressed as [mean ± standard deviation]. To assess the statistical significance, one-way ANOVA followed by Fisher’s LSD post hoc tests were performed. A *p*-value < 0.05 was considered statistically significant.

## Supplementary information

Supplementary Materials

Supple Spreadsheet 1

Supple Spreadsheet 2

Supple Spreadsheet 3

Supple Video 1
