# Supplementary material for: Functionalization of Brain Region-specific Spheroids with Isogenic Microglia-like Cells
Source: Sci Rep. 2019 Jul 30;9:11055. doi: 10.1038/s41598-019-47444-6 (PMC6667451; doi:10.1038/s41598-019-47444-6)
Supplement: Supplementary file 1 — Supplementary Materials [file 41598_2019_47444_MOESM1_ESM.docx]

**Supplementary Materials**

**Functionalization of Brain Region-specific Spheroids with Isogenic Microglia-like Cells**

Liqing Song^1,#^, Xuegang Yuan^1^, Zachary Jones^2^, Cynthia Vied^3^, Yu Miao^1^, Mark Marzano^1^, Thien Hua^4^, Qing-Xiang Amy Sang^4, 5^, Jingjiao Guan^1^, Teng Ma^1^, Yi Zhou^2^, Yan Li^1, 5^ *

^1^Department of Chemical and Biomedical Engineering; FAMU-FSU College of Engineering; Florida State University; Tallahassee, FL USA

^2^Department of Biomedical Sciences, College of Medicine, Florida State University, Tallahassee, Florida, USA

^3^The Translational Science Laboratory, College of Medicine, Florida State University, Tallahassee, Florida, USA

^4^Department of Chemistry and Biochemistry, Florida State University, Tallahassee, Florida, USA

^5^Institute of Molecular Biophysics, Florida State University, Tallahassee, Florida, USA

*Corresponding author:

Dr. Yan Li: address: 2525 Pottsdamer St., Tallahassee, FL 32310, Tel: 850-410-6320; Fax: 850-410-6150; email: [yli@eng.fsu.edu](mailto:yli@eng.fsu.edu).

^#^ Current address: Department of Chemical Engineering; Carnegie Mellon University, Pittsburgh, Pennsylvania, USA

**Supplemental materials include nine figures, one video, three excel spreadsheets, and eight tables.**

**Supplementary Figures**

**Supplementary Figure S1. Microglia differentiation using additional human iPSC line: Ep-iPSC.** Representative fluorescent images of CD45, CD11b, IBA-1, and P2RY12. Blue: Hoechst 33342. Scale bar: 100 μm.

Human Ep-iPSC cells were obtained commercially from ThermoFisher (Cat #A18945). The Gibco Human Episomal iPSC Line was derived from CD34+ cord blood using a three-plasmid, seven-factor (SOKMNLT; SOX2, OCT4 (POU5F1), KLF4, MYC, NANOG, LIN28, and SV40L T antigen) EBNA-based episomal system. This iPSC line is considered to be zero foot-print as there was no integration into the genome from the reprogramming event and is free of all reprogramming genes. Human Ep-iPSC cells were maintained in StemFlex^TM^ Medium (ThermoFisher) on growth factor reduced Geltrex or Matrigel-coated surface. The cells were passaged by Versene (an EDTA-based solution, ThermoFisher) every 3-4 days and seeded at 1:8-1:12 ratio onto the new surface. Microglia differentiation was performed using the same method for iPSK3 line. Preliminary characterization was performed by immunocytochemistry for day 38 cells.

**
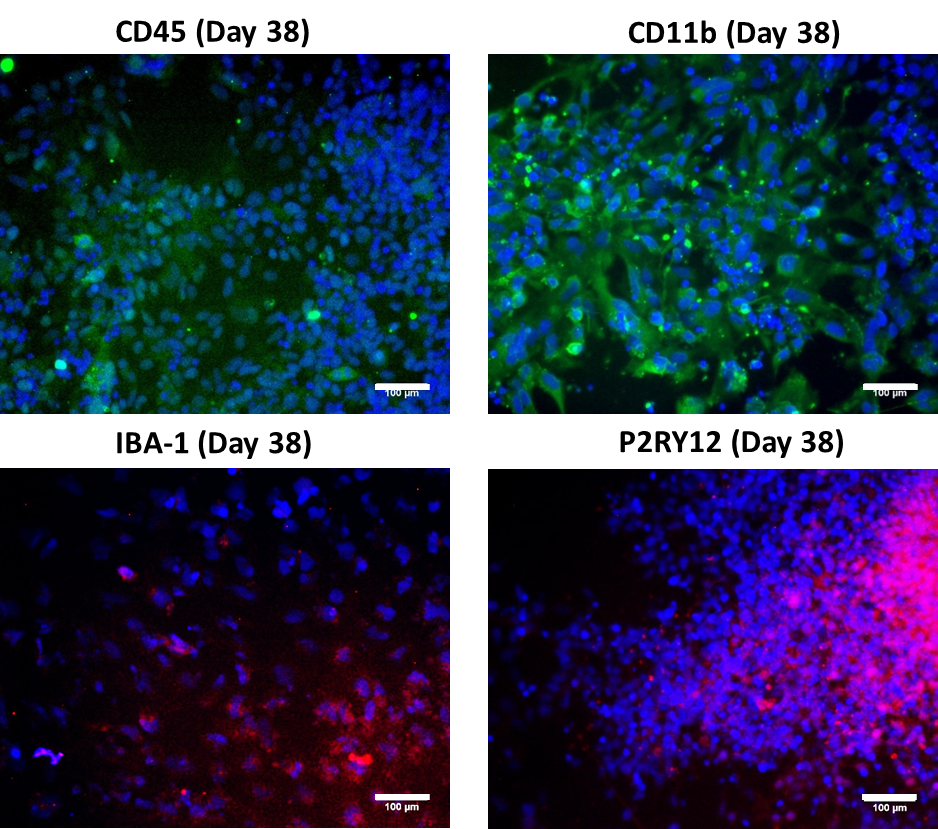
**

**Supplementary Figure S2. Functional characterization of microglia-like cells derived from hiPSCs.** (A) Microglia-like cells phagocytosed fluorescent MPIOs, in comparison to hiPSC-derived neural progenitor cells and hiPSC-derived endothelial cells. The attached microglia-like cells that were not labeled with MPIO served as control. Scale bar: 100 μm. (B) Migration ability of microglia-like cells determined by wound healing assay. (i) Phase contrast images of the injured microglia-like cells; Scale bar: 200 μm. The red arrows show the width decrease of the wound over the time. (ii) Analysis of wound edge migration length. *indicates *p* < 0.05 for the different test conditions.

***Microglia migration assay***

Wound healing assay was modified to evaluate microglia migration (Roney et al., 2011). Briefly, day 35 microglia-likes cell were seed (0.2×10^6^ cell/per well) onto tissue culture treated 24-well plate and grown overnight in DMEM plus 10% FBS. An artificial wound was introduced with a 200 μL pipette tip and images were captured with an Olympus IX70 inverted microscope for 0-24 hours. Change of the area of the scratch over 24 hours was calculated by ImageJ software in terms of pixels, and the rate of migration was calculated as pixels/sec and converted to micrometers/sec. Three wells per group were used for analysis.


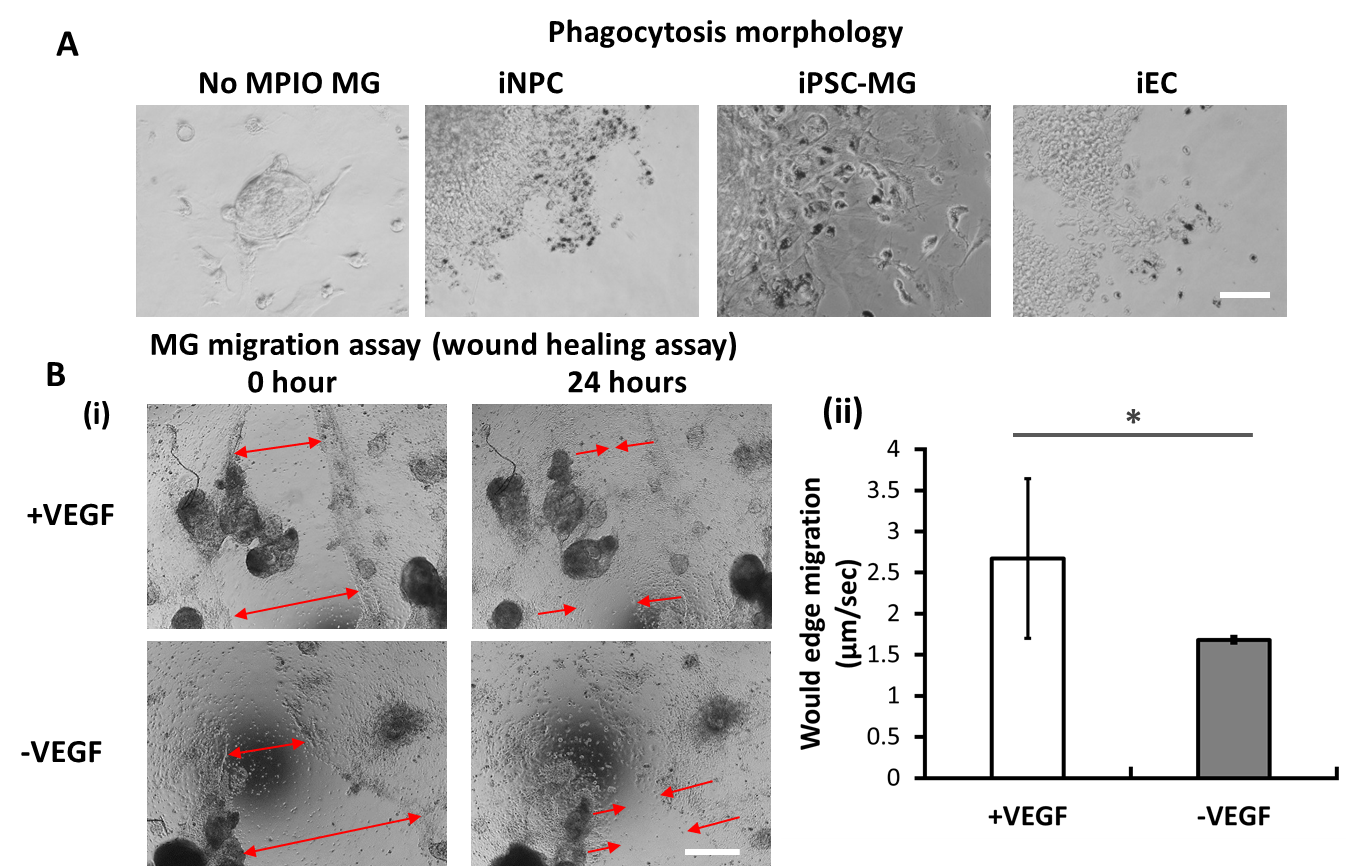


**Supplementary Figure S3. Lipopolysaccharides (LPS) stimulation of microglia-like cells.** mRNA gene expression of (A) TNF-α and IL-6, (B) MMP2 and MMP3 for day 33 microglia-like cells after Aβ42 oligomer stimulation for 72 hours. * and ** indicate *p* < 0.05 for the different test conditions. (C) MMP-9 expression and Aβ42 internalization for microglia-like cells treated with Aβ42 oligomers. Scale bar: 100 μm.


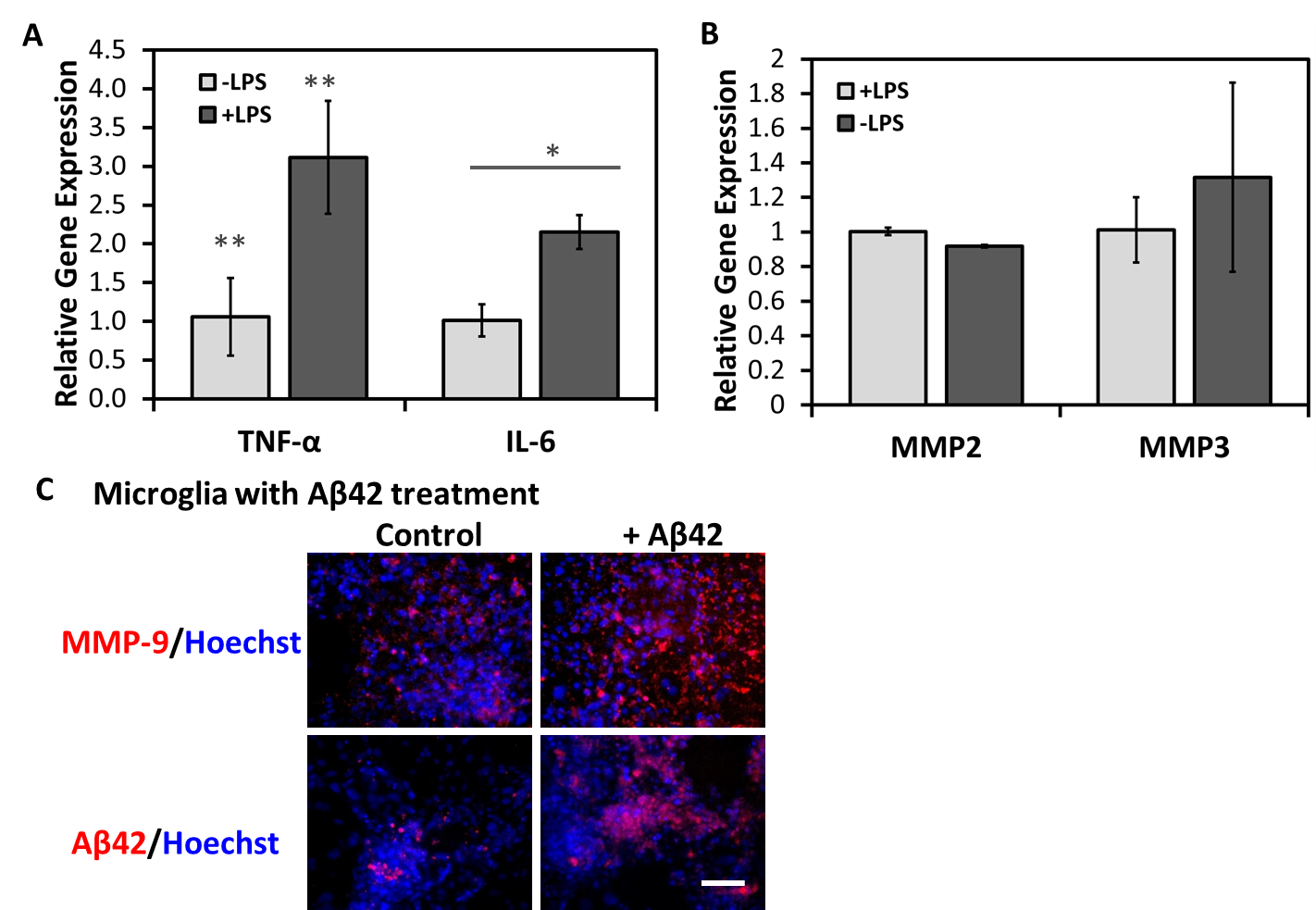


**Supplementary Figure S4. Dorsal and ventral spheroids/organoids generated from hiPSCs in suspension.** (A) Schematic illustration of dorsal and ventral differentiation protocol. (B) Phase contrast images of the derived dorsal and ventral spheroids over 30 days. Scale bar: 400 μm.


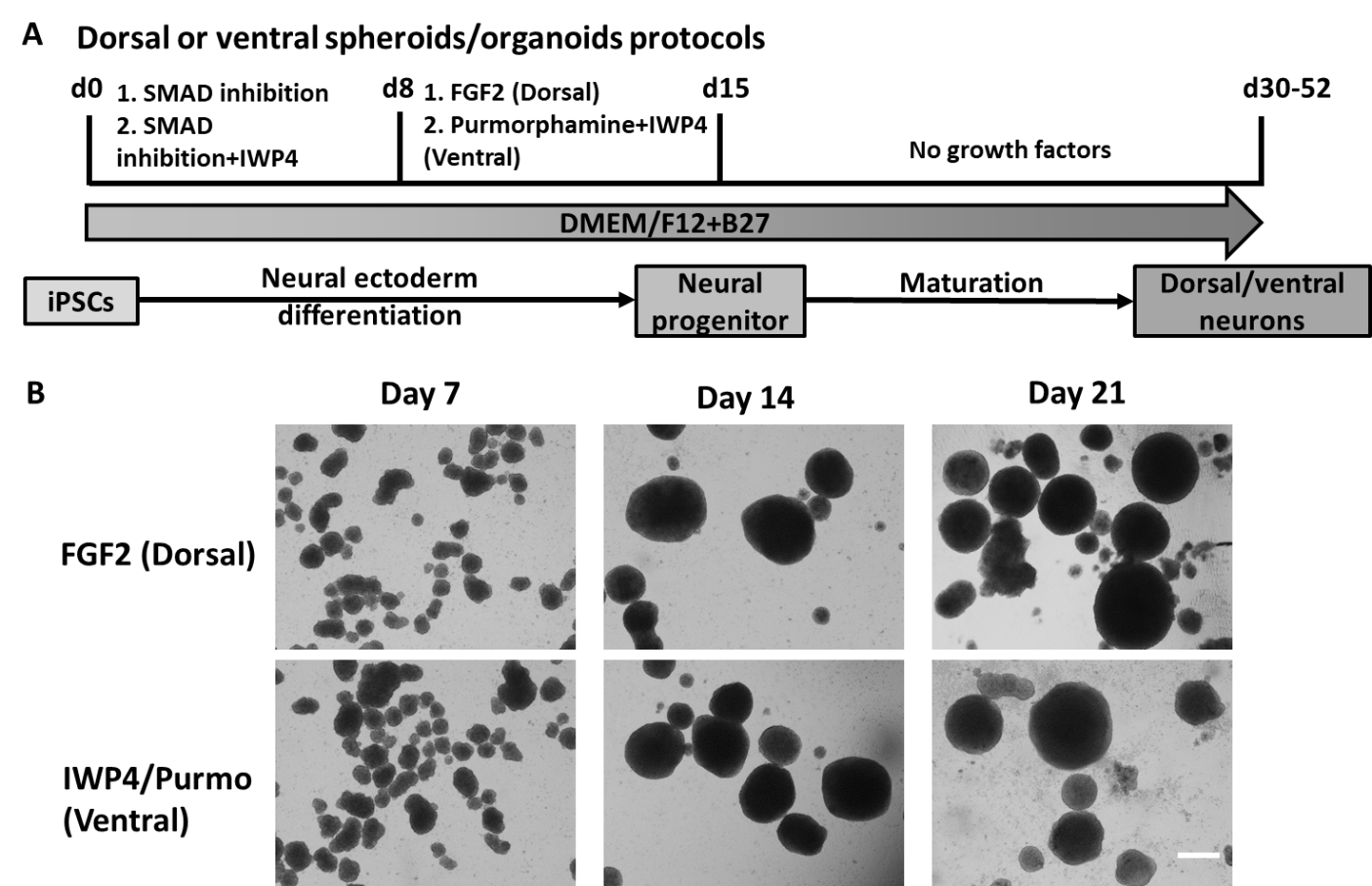


**Supplementary Figure S5. Characterization of dorsal and ventral spheroids derived from hiPSCs in suspension.** Day 30 ventral (IWP4/Purmo) and dorsal (FGF2) spheroids were replated for three days and immunocytochemistry was performed. Representative fluorescent images of neural marker β-tubulin III (green), cortical layer markers, TBR1 (red), SATB2 (green), and BRN2 (red), ventral neuron markers, PAX6 (green) and NKX2.1 (green), Glutamatergic neuron marker, Glut (red), and GABAergic neuron markers, GABA (red) and vGAT (red), for the FGF2 group and IWP4/Purmo group. Blue: Hoechst 33342. Scale bar: 100 μm.


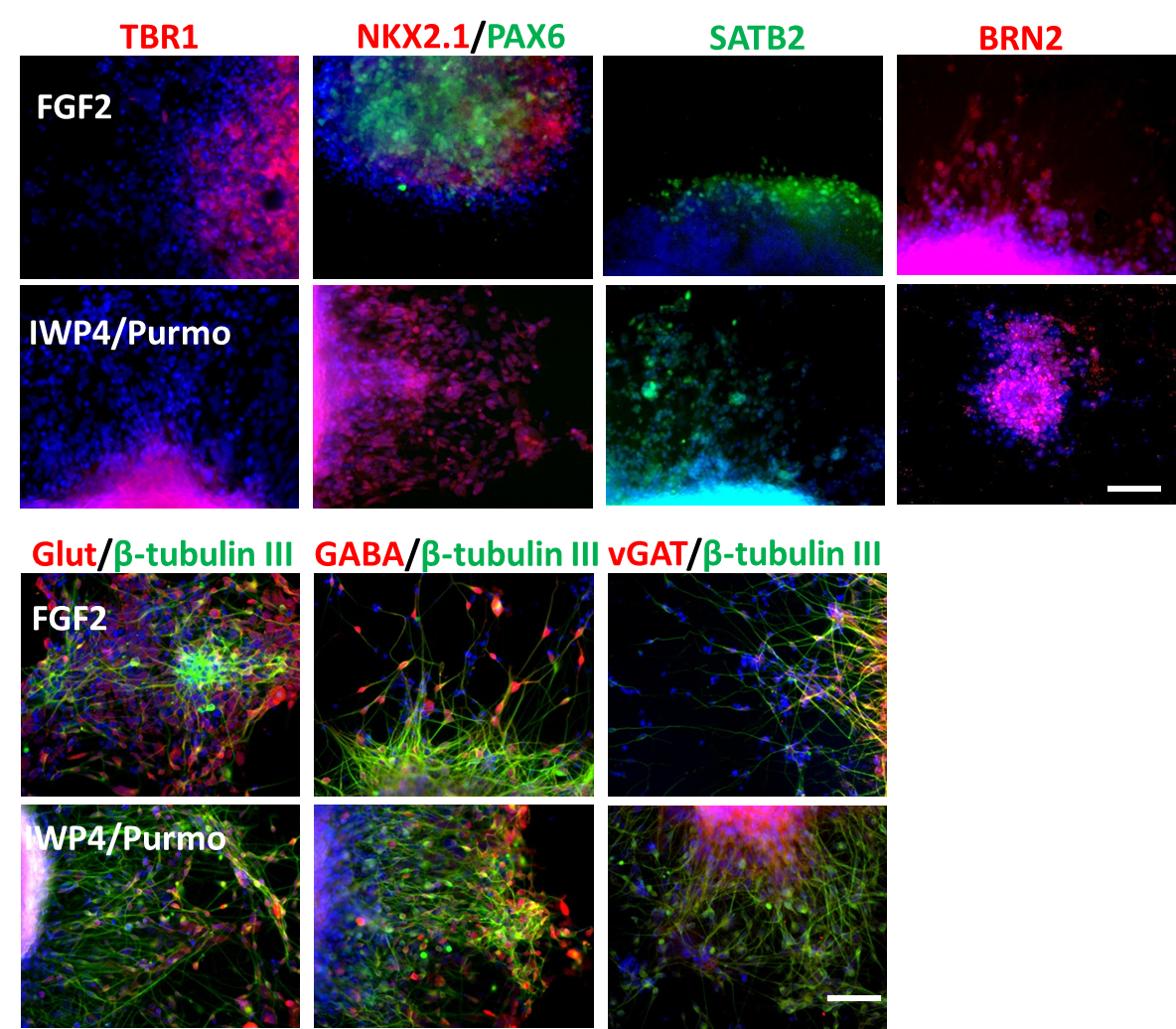


**Supplementary Figure S6. Integration of microglia-like cells with isogenic brain spheroids/organoids.** (A) Phase contrast images of the integration of microglia-like cells (MG) within (i) dorsal spheroids and ventral spheroids at different MG:NPC ratios. Scale bar: 200 μm. (B) Overlay of phase contrast images with fluorescent images to show the integration of microglia-like cells within dorsal spheroids, ventral spheroids; and ventral/dorsal spheroids. Microglia-like cells were labeled with CellTracker Green. Scale bar: 200 μm.

**
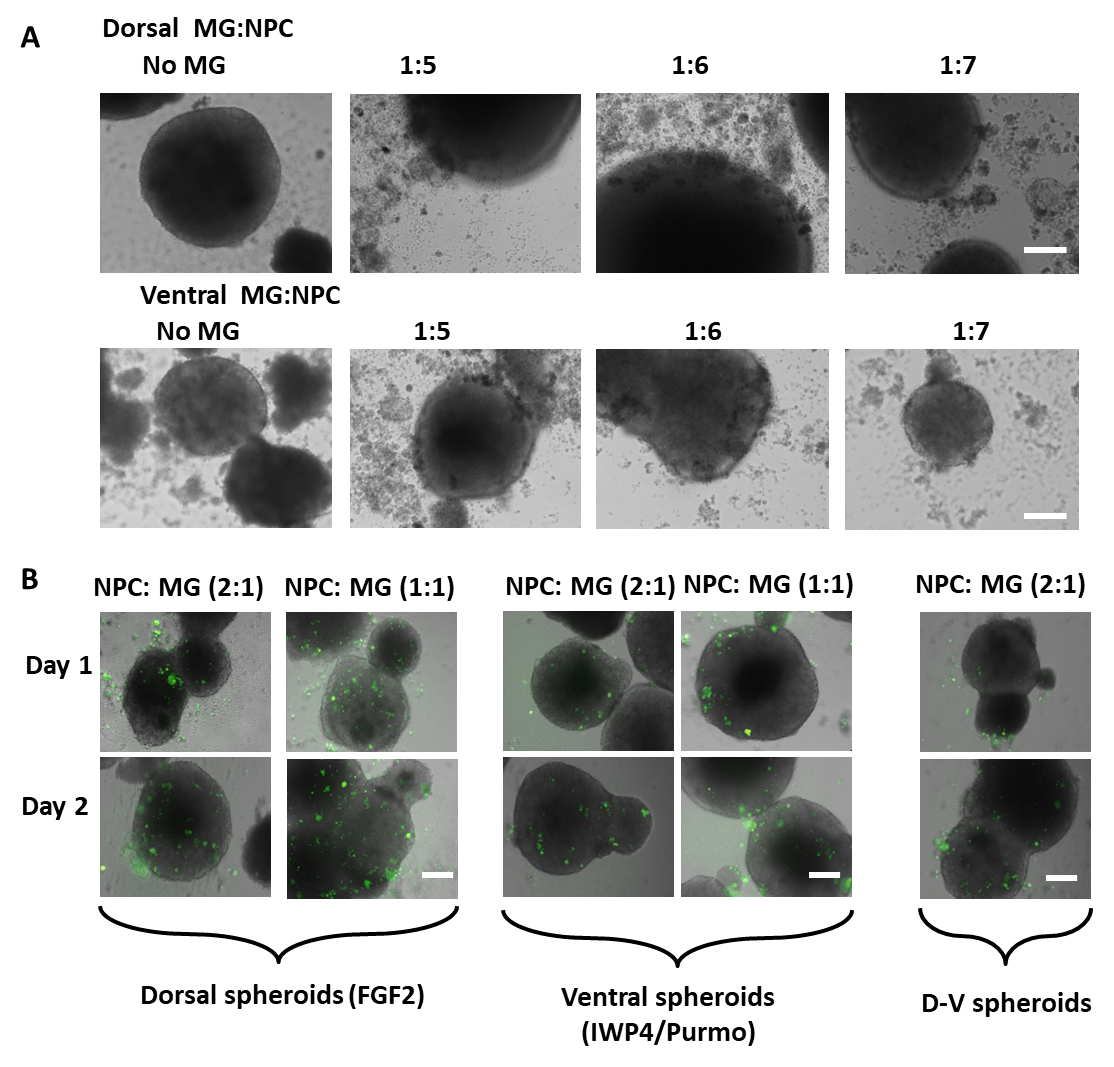
**

**Supplementary Figure S7. Characterizations of histological sections for D-MG and V-MG spheroids (day 47).** (A) H&E staining showed no necrotic center in the spheroids. Scale bar: 200 μm. (B) Immunostaining of histological sections for P2RY12 (red)/β-tubulin III (green) expression. Scale bar: 100 μm.

**
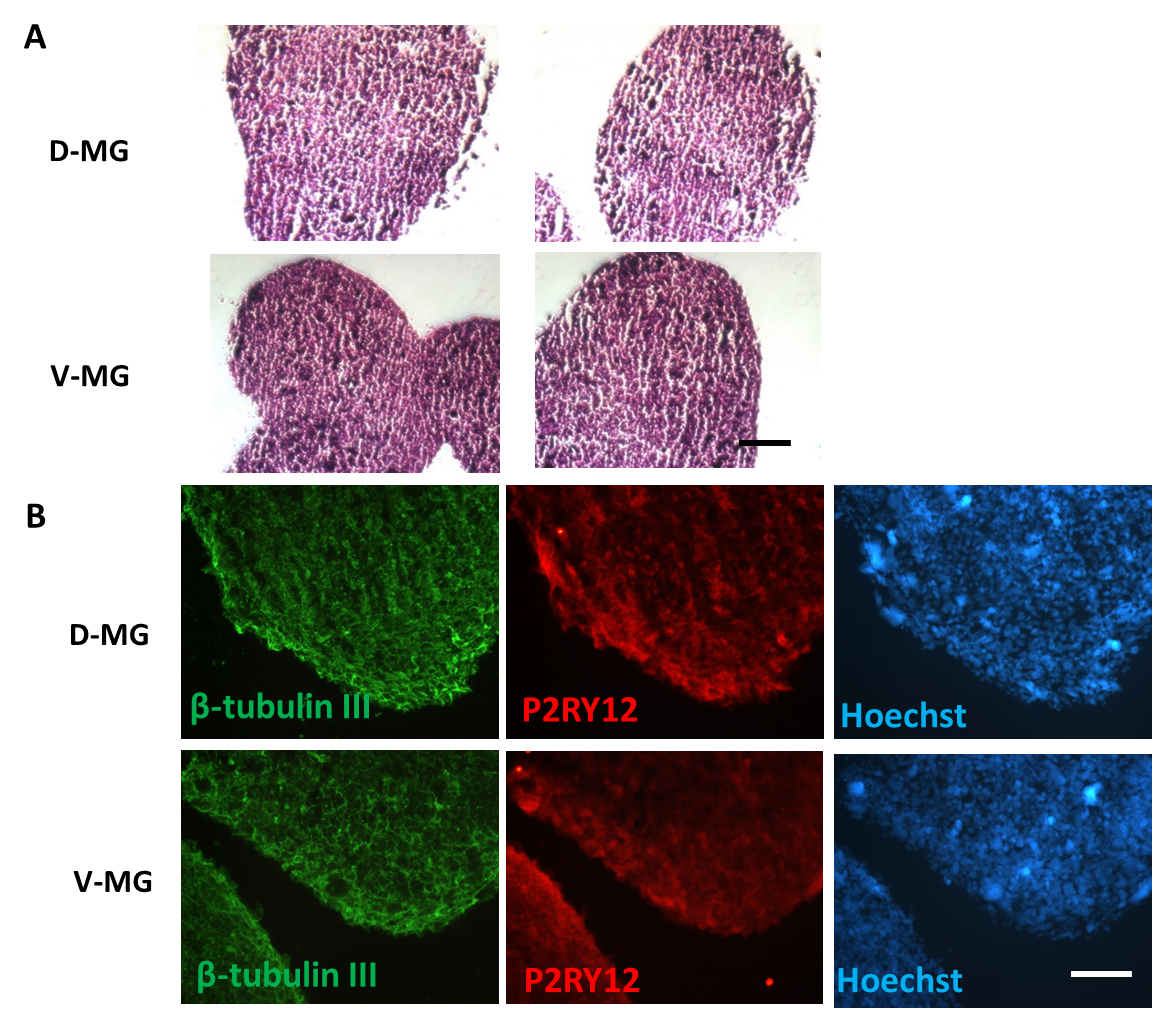
**

**Supplementary Figure S8. Ca^2+^ transient assays (induced by ADP) for dissociated spheroids co-cultured with MGs.** (A) Fluorescent images of Ca^2+^ signaling expression. Scale bar: 100 μm. (B) Quantification of Ca^2+^ fluorescent signals. D-V and MG control groups responded to ADP immediately. More cells from ventral-MG exhibited Ca^2+^ signaling than cells from dorsal-MG group.


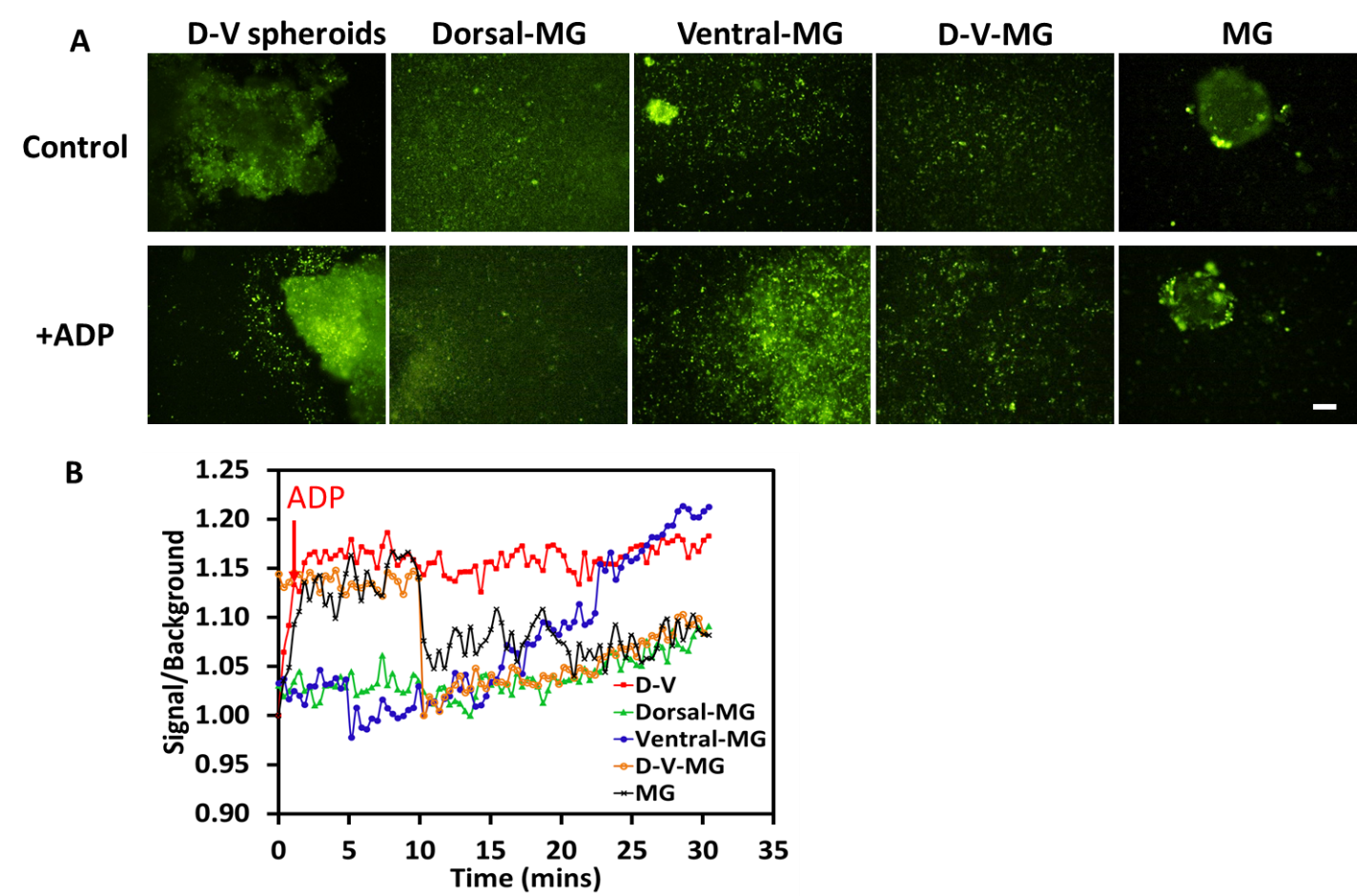


**Supplementary Figure S9. Reactive oxygen species (ROS) expression in co-cultured microglia-like cells and dorsal/ventral organoids.** The day 34 cells were treated with Aβ42 oligomers for 72 hours. (A) Representative fluorescent images of ROS. Scale bar: 100 μm. (B) Quantification of ROS levels by flow cytometry. Black line: negative control; Red line: differentiation in the absence of Aβ42 oligomers; Blue line: differentiation in the presence of Aβ42 oligomers.


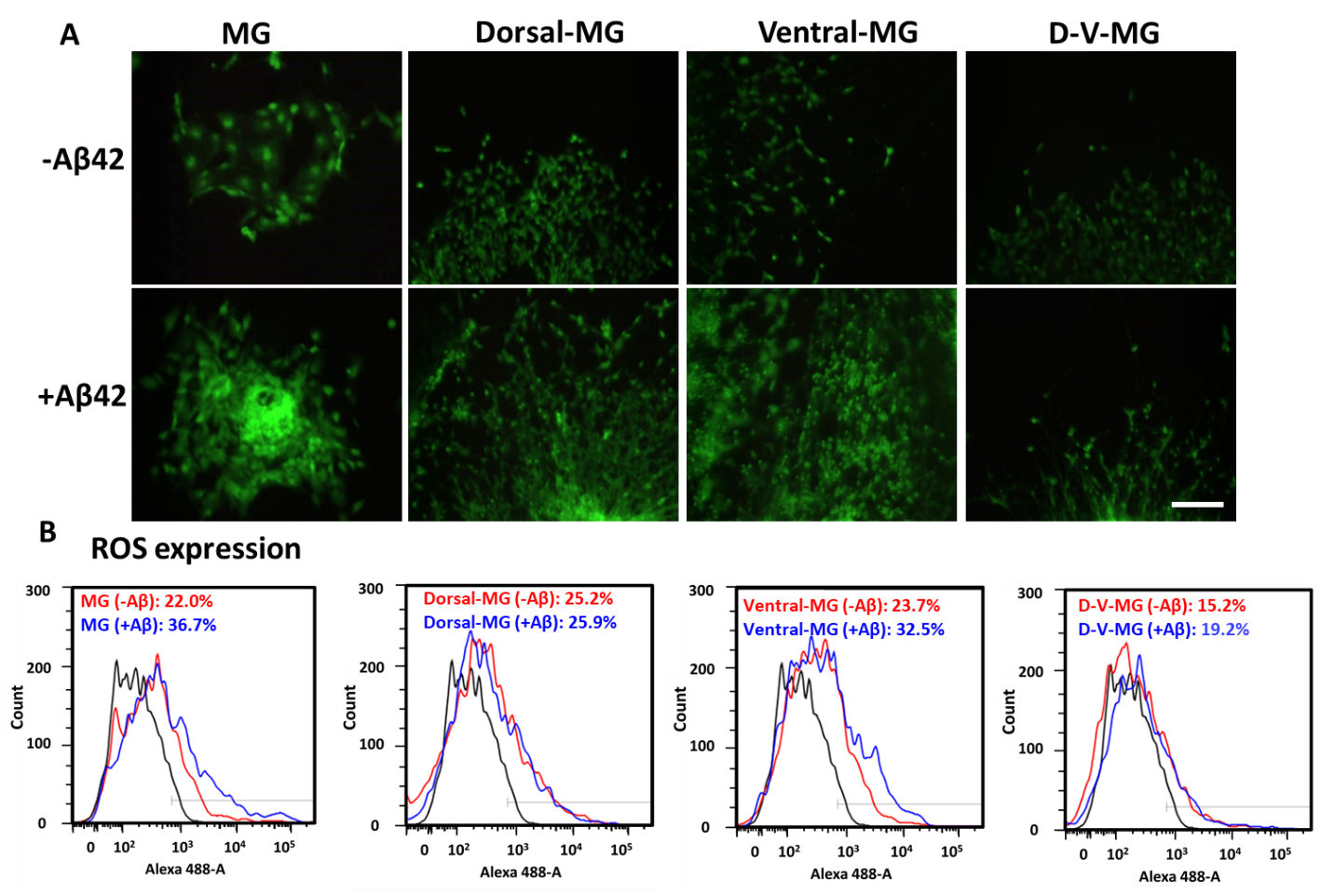


**Supplementary Video 1. Intracellular Ca^2+^ transient expression in microglia-like cells (after adding ADP).**

**Supplementary Spreadsheet 1. “Deseq Up and Down” for RNA-Seq data analysis.**

Sample 1: D-MG. Sample 2: MG

**Supplementary Spreadsheet 2. “GO_500Up_500Down” for RNA-Seq data analysis.**

**Supplementary Spreadsheet 3. “MG_ECM_GO” for RNA-Seq data analysis.**

**Supplementary Table S1. A list of antibodies**

| **Cells** | **Primary Antibody** | **Origin/ Isotype** | **Supplier/ Cat#** | **Dilution** |
| --- | --- | --- | --- | --- |
| Neural cells | PAX6 | Mouse IgG_1_ | Santa Cruz, sc-81649 | 1:100 |
| Cortical layers | TBR1 (layer VI) | Rabbit IgG | ABCAM, ab31940 | 1:200 |
|  | BRN2 (layer III) | Goat IgG | Santa Cruz, sc-6029 | 1:200 |
|  | SATB2 (layer IV) | Mouse IgG1 | ABCAM, ab51502 | 1:5 |
| Neurons | β-tubulin III | Mouse IgG1 | Millipore, MAB1637 | 1:200 |
|  | MAP-2 | Rabbit IgG | ABCAM, ab32454 | 1:200 |
| Ventral | NKX2.1 | Rabbit IgG | ThermoFisher, PA5-25940 | 1:300 |
|  | Glutamate | Rabbit IgG | Sigma, G6642 | 1:1000 |
|  | GABA | Rabbit IgG | Sigma, A2052 | 1:1000 |
|  | vGAT (vesicular GABA transporter) | Rabbit IgG | Sigma, AB2257 | 1:200 |
| Synaptic markers | Synapsin I | Rabbit IgG | Millipore, 574777 | 1:500 |
|  | PSD95 | Rabbit IgG | ThermoFisher, 51-6900 | 1:200 |
| Microglia | KDR | Mouse IgG1 | Millipore, 05-554 | 1:100 |
|  | CD31 | Goat polyclonal IgG | Santa Cruz, sc-1506 | 1:200 |
|  | CD45 | Mouse IgG1 | R&D Systems, MAB1430 | 1:200 |
|  | CD11b | Mouse IgG1 | ThermoFisher, BMS104 | 1:200 |
|  | IBA-1 | Rabbit IgG | ThermoFisher, PA5-27463 | 1:200 |
|  | CX3CR1 | Goat polyclonal IgG | R&D Systems  AF5825-SP | 1:200 |
|  | P2RY12 | Rabbit IgG | Sigma, HPA013796 | 1:200 |
| Proliferation | BrdU | Mouse IgG1 | Life Technologies, 03-3900 | 1:200 |
| Pathway | Aβ 42 | Rabbit IgG | ABCAM, ab10148 | 1:200 |
| ECM remodeling | MMP9 | Goat IgG | Santa Cruz, sc-6840 | 1:200 |
| Secondary | Alexa 488, goat anti-mouse IgG1 | - | Life Technologies,  A-21121 | 1:200 |
|  | Alexa 488, goat anti-rabbit IgG | - | Life Technologies,  A-11034 | 1:200 |
|  | Alexa 594, goat anti-rabbit IgG | - | Life Technologies,  A-11012 | 1:400 |
|  | Alexa 594, donkey anti-goat IgG | - | Life Technologies,  A-11058 | 1:400 |

**Supplementary Table S2. Primer sequence for target genes.**

| Gene | Forward primer 5' to 3' | Reverse primer 5' to 3' |
| --- | --- | --- |
| TNF-a | CCTGGGATTCAGGAATGTGTG | TGTAGGCCCCAGTGAGTTCTG |
| IL-6 | ATGAGGAGACTTGCCTGGTGA | ATCTGCACAGCTCTGGCTTGT |
| MMP2 | CATCGCTCAGATCCGTGGTG | GCATCAATCTTTTCCGGGAGC |
| MMP3 | CCATCTCTTCCTTCAGGCGT | ATGCCTCTTGGGTATCCAGC |
| TBR1 | CCCCCTCGTCTTTCTCTTACC | TAATGTGGAGGCCGAGACTTG |
| PROX1 | GACTTTGAGGTTCCAGAGAGA | TGTAGGCAGTTCGGGGATTTG |
| NKX2.1 | GAGTCCAGAGCCATGTCAGC | GCATAAAACAGCTTTGGGGTGT |
| TREM2 | GTGAAGGAAGATGATGGGAGGA | TTGCCAGAGCAGAACAAGGAG |
| β-actin | GTACTCCGTGTGGATCGGCG | AAGCATTTGCGGTGGACGATGG |

**Supplementary Table S3. A table of GO terms for the bottom 100 genes.**

| ID:GO:0022853 active ion transmembrane transporter activity |
| --- |
| ID:GO:0001098 basal transcription machinery binding |
| ID:GO:0001099 basal RNA polymerase II transcription machinery binding |
| ID:GO:0009055 electron carrier activity |
| ID:GO:0046933 proton-transporting ATP synthase activity, rotational mechanism |
| ID:GO:0015075 ion transmembrane transporter activity |
| ID:GO:0015077 monovalent inorganic cation transmembrane transporter activity |
| ID:GO:0000993 RNA polymerase II core binding |
| ID:GO:0042625 ATPase coupled ion transmembrane transporter activity |
| ID:GO:0022804 active transmembrane transporter activity |
| ID:GO:0008276 protein methyltransferase activity |
| ID:GO:0003700 transcription factor activity, sequence-specific DNA binding |
| ID:GO:0001071 nucleic acid binding transcription factor activity |
| ID:GO:0008324 cation transmembrane transporter activity |
| ID:GO:0046966 thyroid hormone receptor binding |
| ID:GO:0008170 N-methyltransferase activity |
| ID:GO:0043175 RNA polymerase core enzyme binding |
| ID:GO:0022891 substrate-specific transmembrane transporter activity |
| ID:GO:0022890 inorganic cation transmembrane transporter activity |
| ID:GO:0015294 solute:cation symporter activity |

**Supplementary Table S4. A table of expressed genes associated with Alzheimer’s disease.** The FPKM (fragments per kilobase per million reads) normalized values for these genes are listed for both samples. The numbers are the Log2 values of ratios of D-MG to MG. Negative values indicate that the genes are present in higher amounts in MG group, while positive values indicate that the genes are present in higher amounts in D-MG group.

**Supplementary Table S5. A table of expressed genes associated with cortical neurons.**

**Supplementary Table S6. A table of expressed genes associated with oligodendrocytes.**

**Supplementary Table S7. A table of expressed genes associated with astrocytes.**

**Supplementary Table S8. A table of expressed genes associated with brain pericytes.**

**Reference:**

Roney, K.E., O'Connor, B.P., Wen, H., Holl, E.K., Guthrie, E.H., Davis, B.K., Jones, S.W., Jha, S., Sharek, L., Garcia-Mata, R., Bear, J.E., Ting, J.P., (2011) Plexin-B2 negatively regulates macrophage motility, Rac, and Cdc42 activation. PLoS One 6, e24795.
